# Formalising privacy regulations with bigraphs

**DOI:** 10.1007/s10270-025-01293-2

**Published:** 2025-06-24

**Authors:** Ebtihal Althubiti, Blair Archibald, Michele Sevegnani

**Affiliations:** 1 Computer Science Department, Northern Border University, 91431 Arar, Saudi Arabia; 2 School of Computing Science, University of Glasgow, Glasgow, Scotland, G12 8QQ UK

**Keywords:** Privacy regulations, Privacy modelling, Formal modelling, Model checking, Bigraphs

## Abstract

With many governments regulating the handling of user data—the General Data Protection Regulation, the California Consumer Privacy Act, and the Saudi Arabian Personal Data Protection Law—ensuring systems comply with data privacy legislation is of high importance. Checking compliance is a tricky process and often includes many manual elements. We propose that formal methods, that model systems mathematically, can provide strong guarantees to help companies *prove* their adherence to legislation. To increase usability we advocate a diagrammatic approach, based on bigraphical reactive systems, where privacy experts can explicitly *visualise* the systems and describe updates, via rewrite rules, that describe system behaviour. The rewrite rules allow flexibility in integrating privacy policies with user-specified systems. We focus on modelling notions of *providing consent, withdrawing consent, purpose limitations, the right to access and sharing data with third parties*, and define privacy properties that we want to prove within the systems. Properties are expressed using the computation tree logic and proved using model checking. To show the generality of the proposed framework, we apply it to two examples: a bank notification system, inspired by Monzo’s privacy policy, and a cloud-based home healthcare system based on the Fitbit app’s privacy policy.

## Introduction

Enhancements in sectors including education, governance, healthcare, and finance [1], depend on sensing and data aggregation techniques to collect information about users. The amount and types of personal data collected make privacy a significant concern [2], and even collecting only non-personal information presents privacy risks through the ability to predict sensitive information about individuals [3].

To alleviate privacy concerns, governments have imposed regulations that protect users’ private information and clarify their rights. A large number of regulations exist, including the Australian Privacy Principles (APPs) [4], the European Union (EU) General Data Protection Regulation (GDPR) [5], the California Consumer Privacy Act (CCPA) [6], the Saudi Arabian Personal Data Protection Law (PDPL) [7], the Georgia Computer Data Privacy Act (GCDPA) [8], and the American Data Privacy and Protection Act (ADPPA) [9].

Failing to adhere to these regulations can result in fines for organisations. For example, based on the GDPR, organisations may face fines of up to 4% of their global annual income or 20 million euro, whichever is higher [10].

Given the range of regulations, how do organisations ensure information systems are robustly designed to avoid privacy violations? This issue is further compounded by the fact the regulations are non-formalised, e.g. textual, subject to change, and written by and for lawyers rather than system designers [11].

**Fig. 1:**
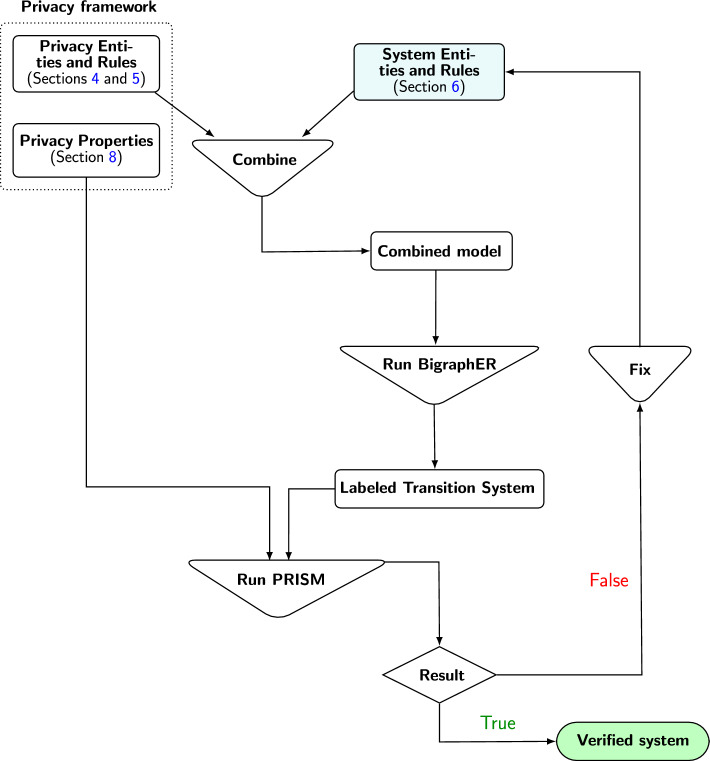
Bigraph-based privacy framework: the teal box represents the user-specified elements. These elements are combined with the privacy rules and entities to form a unified model. BigraphER then analyses this unified model to generate a labelled transition system. The model is subsequently verified using the PRISM model checker to assess its compliance with the specified privacy properties. If the verification result is positive, the system is considered to meet the privacy properties. If the result is negative, the system must be revised to address the identified issues, after which it undergoes reanalysis. This iterative process continues until the system successfully passes verification

We believe formal methods can play a key role in enabling companies to prove their compliance to privacy legislation. Formal methods are techniques to model, analyse, and verify systems’ specifications mathematically and allow guarantees/proofs of, for example, correctness, safety, and security [12]. Formal methods allow a shift away from text-based regulations and into a form amenable to exhaustive verification via automatic tooling, while formal modelling approaches have been proposed to check compliance with privacy regulations [14, 15, 51], the majority only model GDPR, and a limited subset, i.e. providing/withdrawing consent or defining purposes. None of these approaches currently consider regulations restricting cross-border data transferring as they lack support for spatial properties. They also often require significant updates when privacy policies change as they are typically defined in terms of a fixed set of rules.

We propose a novel approach to privacy modelling based on Milner’s bigraphs [16]: a computational model that is visual in nature, and that specifies systems based on the spatial and non-spatial relationships between entities. Bigraphs can evolve over time through rewriting using reaction rules, allowing privacy updates to be modelled e.g. to describe consent being given or withdrawn, and data movement. Bigraphs have several benefits: (1) they are flexible, as entities and reaction rules are user-specified, e.g. the same formalism can capture financial and healthcare domains; (2) reaction rules enable us to extend and amend models easily based on the changes that may occur in the privacy regulations or the underlying system; (3) they have a diagrammatic notation, not unlike you might draw on a whiteboard, that is suitable for system designers to understand and describe the model; (4) they natively express spatial properties, e.g. containment relation, that enables us to model the GDPR requirements for cross-border data transfer; (5) they allow multi-perspective modelling where privacy concerns can be modelled independently of a specific system but interact with it via explicit links.

Figure 1 summarises the proposed framework. The framework consists of pre-defined (but modifiable) privacy entities and reaction rules. Users define system entities, using bigraphs, tailored to their domain (e.g. a banking system). The privacy entities can be customised by adding or removing entities as needed. Once linked to the system entities, users define the system’s reaction rules and integrate them with the privacy reaction rules to model privacy regulations. Users can further customise the privacy reaction rules by selecting and removing unnecessary reaction rules. The combined model, consisting of both system and privacy entities along with their reaction rules, is executable using the BigraphER tool [17]. The framework also provides an extendable set of privacy properties that should be verified e.g. providing consent. These privacy properties are expressed using the Computation Tree Logic (CTL) and can be *automatically* verified using PRISM [18]. Although these properties are predefined, end-users must specify system-related aspects, e.g. sharing data with a third-party system entity.

To prove the effectiveness of our approach, we apply it to *two* example systems. Although we abstract away certain system-specific aspects, the framework is comprehensive enough to capture the privacy regulations and express the privacy violation properties discussed in Sect. 8. Our goal is to show how the privacy model is constructed and utilised, rather than how to build a good system model (which is largely independent of the privacy perspective; see Fig. 1).

We believe the approach can be used by a variety of end-users to prove their systems’ compliance with several privacy regulations. It is particularly applicable to those regulations with notions of *providing consent, withdrawing consent, purpose limitations, the right to access, and sharing data with third parties*. These notions are key principles required by privacy regulations to give users control over their data [19]. The framework’s end-users benefit from the diagrammatic notation to help explain the model to privacy experts, e.g. privacy and data-protection lawyers.

We make the following research contributions:

- We construct a bigraph-based framework for privacy. The model allows notions of providing/withdrawing consent, purposes limitation, the right to access and sharing data with third parties, all of which are required by most privacy regulations including GDPR and CCPA. We leverage the inherent spatial modelling capabilities of bigraphs to explicitly capture cross-border data transfers and support rigorous spatial verification.
- We demonstrate the applicability of our framework by applying it to two examples: a bank notification system based on GDPR requirements and a cloud-based home healthcare system example based on CCPA requirements.
- We identify common privacy properties and show how the model, combined with model checking, enables these to be formally checked.

The paper is structured as follows. Section 2 introduces the key elements of the privacy policies we want to model, and Section 3 introduces the theory of bigraphs and bigraphical reactive systems. Section 4 explains how we model system privacy based on a multi-perspective approach, while Sect. 5 shows how, through reaction rules, we model the dynamic nature of privacy, e.g. performing permission checking. The integration between the privacy framework and system-specific aspects is explained in Sect. 6. We show the approach is reusable in Sect. 7 by applying it to a second system example, and in Sect. 8 explain how the formal model unlocks the ability for formal privacy policy verification through model checking. We highlight the features and the limitations of the framework in Sect. 9. Related work is in Sects. 10 and 11 concludes.

## Privacy regulations

There is no universally agreed-upon definition of privacy as it depends on individuals’ cultures and governments’ rules [13]. Solove proposes a widely accepted taxonomy [20] that categorises privacy violations into four groups: invasion violations (e.g. stealing a USB flash drive), information collection violations (e.g. surveillance without consent), information processing violations (e.g. using data for unintended purposes or preventing users from accessing their data), and information dissemination violations (e.g. unauthorised sharing of user data). As handling invasion violations requires specifying a full physical security model, we only focus on the last three categories.

Privacy regulations like the APPs, GDPR, PDPL, GCDPA, and ADPPA require organisations to obtain user consent before handling their data. The CCPA introduces the concept of *notice at collection* [6] which assumes user agreement by default. Obtaining user consent or informing them about data collection can avoid information collection violations. These regulations also grant users rights to access and withdraw consent, aiming to prevent information processing violations [6–9, 21, 22]. These regulations impose measures to prevent information dissemination violations, such as protecting data from unauthorised access and restricting the sharing of data with third parties [6–9, 23, 24].

The GDPR is one of the most stringent regulations [25], imposing specific legal bases for data transfers to non-EU countries [26]. One of the legal bases for such transfers is the adoption of Standard Contractual Clauses (SCCs), a set of pre-defined rules approved by the European Commission that must be included in contracts between data senders and receivers. In this paper, we focus on modelling SCCs, while other legal bases are introduced in [27].

As all these regulations share common privacy requirements, it is possible to develop a unified framework to detect violations and formally prove compliance.

## Bigraphical reactive systems

Bigraphical reactive systems (BRSs) [16] are a universal model of computation that describes systems based on how entities change their connectivity and locality (placement) as the system evolves. A key benefit of BRSs is that they have an equivalent algebraic and diagrammatic (visual) notation allowing them to be used by those without a formal background in mathematics [28, 29] without sacrificing mathematical rigour, e.g. their ability to prove system properties. In this paper, we focus on the diagrammatic notation, but full (equivalent) algebraic models are available online [30].

**Fig. 2:**
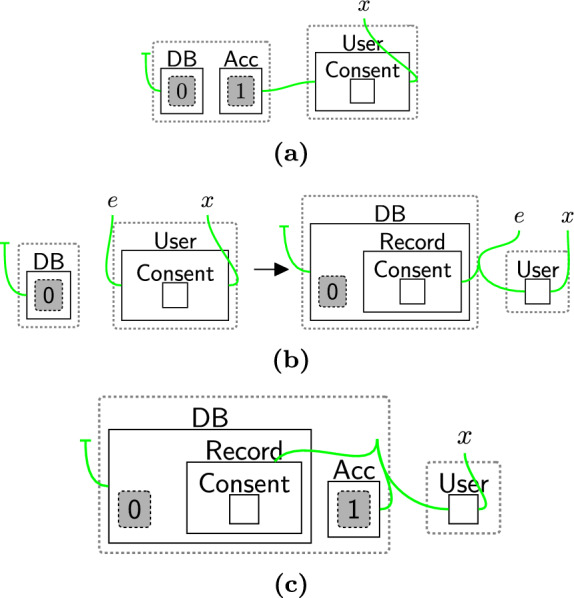
**a** A bigraph modelling a database and account (with some abstracted contents) and a user wanting to register consent; **b** a rule allowing a user to pass consent to a database. The names allow any additional connections to the user; **c** the result of applying rule (**b**) to bigraph (**a**): the consent is moved from the user and added as a record in the database

We introduce bigraphs by example. Figure 2a is a bigraph representing a $\textsf{User}$ that has some $\textsf{Consent}$—shown using *nesting*—a database ($\textsf{DB}$), and an account ($\textsf{Acc}$). Shapes denote different entities, and we sometimes distinguish entities using colours. Grey dashed rectangles are *sites* that represent entities/bigraphs that have been abstracted away, that is, an unspecified bigraph can be placed there. For example, site 0 will have database-specific information stored in it. The dashed unfilled rectangles are *regions* that represent (disjoint) areas of the system, e.g. the database storing the user information exists somewhere else separate to the user. Note: we do not need to say *where* it is, only that it is somewhere else. Parallel regions are often used to model *perspectives* [29] where different concerns are modelled independently, e.g. privacy and systems concerns. We discuss this further in Sect. 4.

Green (hyper)links represent non-spatial relationships between entities. Open links connect to names, e.g.  *x*, that, like sites, specify this link *might* connect elsewhere. A closed link, e.g. the link connecting $\textsf{User}$ with $\textsf{Record}$ and $\textsf{Acc}$ in Fig. 2c, only connects these entities. Closed links may be one−to−zero hyperedges, e.g. the link attached to $\textsf{DB}$. For ease of readability, we sometimes colour links connecting different types of entities.

The corresponding algebraic form of the bigraph in Fig. 2a is:

$$\begin{aligned} \mathsf {/e \ ((/y \ DB_y.id \mid Acc_e.id ) \parallel User_{e,x}.Consent}) \end{aligned}$$

For a detailed explanation of the algebraic notations used, refer to Table 1.

**Table 1:** Description of algebraic notations used in bigraphical reactive systems (BRSs)

Algebraic notation	Term	Diagrammatic notation
$\textsf{id}$	Identity	
$\mathsf {User_{e,x}.Consent}$	Nesting	
$\mathsf {/y \ DB_y.id}$	Link closure	
$\mathsf {/e \ (Acc_e.id \parallel User_{e,x}.id )}$	Parallel Product	
$\mathsf {/y \ DB_y.id \mid Acc_e.id}$	Merge Product	

We specify how bigraphs can evolve over time by using reaction rules. Throughout the paper we use rule to mean reaction rule. Each rule, , consists of a left-hand side (*L*), representing the pattern that will be changed, and a right-hand side (*R*), representing the replacement. An example rule is in Fig. 2b . This rule moves the $\textsf{Consent}$ of a $\textsf{User}$ to a linked $\textsf{DB}$. Note this link is only to identify the database and is not required for movement. The result of applying this rule to the bigraph in Fig. 2a is in Fig. 2c.

Sites and names are particularly important since they let us hide the parts of the model not involved in the rewriting. Importantly, specific names do not matter in reaction rules as they are only used to identify links that may connect elsewhere.

Instantiation maps may be defined for rules to allow copying, swapping, or deletion of sites when a rule is applied. In our notation, we number sites on the left, and their positions after rule application are numbered right-hand sites. Sites that appear on the left but not the right are deleted. For example, in Fig. 2b we use the identity map that sends site 0 on the left to site 0 on the right.

To analyse a BRS we use BigraphER [17], an open-source tool for modelling, rewriting, and visualising bigraphs.

BigraphER enforces the ordering of rules through priority classes, where each class is a set of rules. Suppose we have two classes: $P_1$ and $P_2$, with $P_1$ having a lower priority than $P_2$. In this case, a rule in class $P_1$ can be applied only if no rules in class $P_2$ can been applied.

BigraphER also supports parameterised entities and rules. For example, we can define entities like $\textsf{User}$(*x*) where *x* is drawn from a set of integers or strings, e.g. $x \in \{\texttt{ID}, \texttt{Name} \}$. This is equivalent to defining a set of entities, e.g. $\mathsf {User\_ID}$, $\mathsf {User\_Name}$, .... Rules may be parameterised in a similar fashion.

We also use conditional bigraphs [31], which allow rules to only apply in specific contexts. These are written after a rule in angle-bracket notation. For example,  where − indicates a negative condition (should not exist), $\textsf{True}$ entity is the bigraph^1^ we want to disallow, and $\downarrow$ indicates we should disallow this inside the sites. We also allow positive $+$ (must exist), and contextual conditions $\uparrow$ (anywhere other than the sites/match). We use bigraphs to mean conditional bigraphs throughout.

## A visual approach to privacy modelling

We now define our privacy model. A key goal is to separate the user-defined systems, e.g. a banking application, from the specific privacy policies in order to reuse as much of the model as possible in future scenarios. To enable this, we make use of multi-perspective modelling, where specific aspects of the system appear in their own regions. This approach has been used to good effect in [32–34].

We describe our modelling approach using an example of a bank notification system inspired by Monzo Bank’s privacy policy [35]. A user generates transactions using the Monzo mobile app. Monzo stores the user name and transaction details to calculate the total amount spent, and sends it as a push notification to the user. The bank can share transaction information with a third-party advertiser, e.g. *AnalogFolk company* [36], **if** the user has given consent. The parent company of the third party is located in the $\texttt{UK}$, and it needs to share transaction information with its branch in $\texttt{China}$ [37]. We model from the view of a *single* user interacting with the system, as the data processing is equivalent for all users. This means, however, we cannot model, for example, issues where data is sent to the wrong user.

**Fig. 3:**
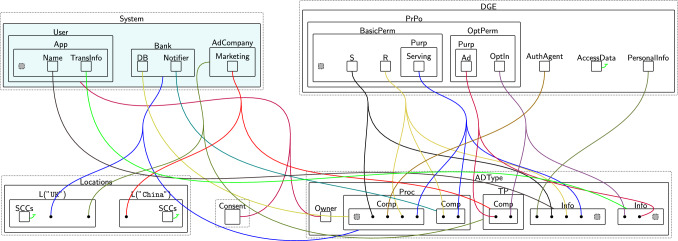
Partial initial bigraph (state) for the banking system scenario. System-specific entities are in teal, while uncoloured regions are part of the reusable privacy model. The perspectives are referred to by the names of their top-level entities: $\textsf{DGE}$ perspective, $\textsf{ADTypes}$, $\textsf{Locations}$ perspective, and $\textsf{Consent}$ perspective. Solid black bullet points are entities (referred to as pointers) that allow end-users to assign an arbitrary number of links. Each system entity is linked to its corresponding type in $\textsf{ADTypes}$, its location, and its permissions in $\textsf{DGE}$. For example, $\textsf{Marketing}$ is linked to $\textsf{Comp}$, which is nested within $\textsf{TP}$, and its location in $\texttt{China}$. To link $\textsf{Marketing}$ with its permissions, we connect the pointers nested within $\textsf{Comp}$ to $\textsf{OptIn}$ and the purpose of accessing the user’s data, which is $\textsf{Ad}$. The site nested within $\textsf{BasicPerm}$ represents the update permission ($\textsf{U}$), while the site nested within $\textsf{App}$ represents the user request to start using the system ($\textsf{ReqUseSys}$). The other sites are pointers linked to either the update permission ($\textsf{U}$) or the read permission ($\textsf{R}$)

Figure 3 shows a partial model of the banking system with the system-specific entities in teal^2^. It consists of a database ($\textsf{DB}$), a $\textsf{Notifier}$ and an $\textsf{AdCompany}$. The $\textsf{Notifier}$ reads the stored transactions, calculates the total amount spent, and sends it as a push notification to the $\textsf{User}$ through the Monzo mobile $\textsf{App}$. The $\textsf{AdCompany}$ is a third party that gathers the user’s transaction information, i.e. $\textsf{TransInfo}$, for advertisement purposes. $\textsf{Marketing}$ is the marketing branch of $\textsf{AdCompany}$ with which $\textsf{AdCompany}$ needs to share the data. We use the general term *agent* to refer to system entities, e.g. $\textsf{DB}$ and $\textsf{Notifier}$.

To use these system-specific entities and data with the privacy model, they must be mapped to general privacy types, e.g. data processors, third parties, information, etc. This is handled by the $\textsf{ADTypes}$ perspective (Fig. 3, Bottom right). The agents’ physical location must also be specified by linking each agent to an $\textsf{L}$ entity in the $\textsf{Locations}$ perspective. The specific policies applied to different entities and data types are then provided in the $\textsf{DGE}$ perspective keeping the general purpose privacy policies separate from the system being regulated. Finally, the $\textsf{Consent}$ perspective keeps track of the granted permissions and the accepted purposes as we will discuss in Sects. 4.4 and 5.1.

**Table 2:** DGE perspective entities

Entity	Description
$\textsf{PrPo}$	The privacy policies that are determined by the DGE: either basic permissions ($\textsf{B}$asicPerm) or optional permissions ($\textsf{O}$ptPerm)
$\textsf{AuthAgent}$	Linked with in order to determine Authorised agents
$\textsf{PersonalInfo}$	Whether information needs stronger privacy
$\textsf{AccessData}$	Linked to when the user has a right to access data

### Modelling data governance entity

*Data controllers* under the GDPR and *businesses* under the CCPA [6, 38] are responsible for determining privacy policies, e.g. who has access to data and with what permissions [6, 39]. We use the term *Data Governance Entity* ($\textsf{DGE}$) to generalise across the GDPR and CCPA regulations. The entities of this perspective are listed in Table 2.

We identify these parties and data types using hyperlinks as *sets*. For example, an $\textsf{AuthAgent}$ links to all agents in the $\textsf{ADTypes}$ perspective that can access personal information. Conversely, if the entity is not linked to $\textsf{AuthAgent}$, then it is unauthorised. $\textsf{PersonalInfo}$ similarly identifies *sets* of data through linking. The classification of information into personal and general is based on the definition of the personal information in the privacy regulation that we aim to model. For example, GDPR and CCPA classify any information that can *lead to* identification of a specific person as personal. The aim of specifying authorised entities and personal information is to prove there is no unauthorised access to personal data (see Sect. 8).

A core entity is the Privacy Policies ($\textsf{PrPo}$) which are either $\textsf{BasicPerm}$ or $\textsf{OptPerm}$. $\textsf{BasicPerm}$ contains the permissions the system **needs** consent for in order to provide its services to the user. Basic permissions are *store* ($\textsf{S}$) and *read* ($\textsf{R}$). The basic purpose ($\textsf{Purp}$) is $\textsf{Serving}$, i.e. serving the system’s users. End-users of the model can add additional permissions and purposes according to their needs, e.g. writing permission and research purpose. $\textsf{OptPerm}$ contains permissions that are not essential to provide services to the users, e.g. advertising only. Even if a user rejects these, the user is still able to use the system. Here we have additional, but extensible, permissions, and purposes, including the $\textsf{OptIn}$ permission, and $\textsf{Ad}$ (advertising) purpose.

During modelling, hyperedges are added to link *each* permission/purpose with: (1) the data that needs this permission, and (2) the entity that performs this processing.

$\textsf{AccessData}$ is used to denote when an owner can access their data. This is described in detail in Sect. 5.3.

### Specifying agent and data types

Agents and data types in privacy regulations are defined in abstract terms, e.g. a processor. To map these to system-specific agents or data, e.g. a database, we use an $\textsf{ADTypes}$ perspective that maps, using links, system agents, and data to general regulatory agents/terms. This provides flexibility in, for example, mapping the same system term to different agent and data types based on the privacy regulation being modelled and decoupling privacy rules from system rules.

We support the common agent and data types specified in Table 3, but the model is extensible and more could be added if necessary for a specific system/regulation pair, e.g. subprocessor.

**Table 3:** Supported agent and data (AD) types

ADType	Description
$\textsf{Owner}$	The agent who owns the data: usually a user
$\textsf{Proc}$	Processor, the agent that **needs** to process data to provide a service according to the controller’s instructions
$\textsf{TP}$	Third party, other agents who may hold user data for a specific purpose
$\textsf{Info}$	Specific data being managed

Owners represent agents who own specific data, which usually corresponds to the users of the system^3^. Agents acting as owners get their own $\textsf{Owner}$ entity within the $\textsf{ADTypes}$ region, and the relationship is tracked through links. Data owners have specific operations with regard to consent, and these are discussed in Sects. 5.1, 5.3, and 5.5.

Data processors are responsible for the safe handling of owners data to fulfil a particular service. In the banking example, $\textsf{Bank}$ is the main data processor and multiple subcomponents, e.g. databases, may fall under the remit of this main processor. We model this through nesting, with a $\textsf{Proc}$ entity containing multiple processor components $\textsf{Comp}$. These components may have their own authorisation level and permissions. Each $\textsf{Comp}$ is linked to the system entity that processes the data (e.g. $\textsf{DB}$), the authentication level (if it is authorised), and the component’s permissions.

As bigraph entities have a fixed number of ports, we use additional entities, denoted by solid black bullet points, to allow any number of links to be specified. For simplicity, we say these agents are linked to each other, even though there is an additional level of indirection in practice.

Third parties are similar to data processors, but have reduced access to owner data. We model them in a similar way: with a $\textsf{TP}$ entity nesting any subcomponents of the specific third party.

Finally, we abstract specific information, e.g. a users name, to a general $\textsf{Info}$ data type. $\textsf{Info}$ is linked with the specific properties we want of that data, for example, is it personal or general, and what permissions (i.e. store/read/update) does it have.

### Specifying agent locations

Each agent is linked to a Locations in the $$\textsf{Locations}$$ perspective (Fig. 3, bottom left). This allows checking the GDPR requirements for sharing data with third parties outside the EU. A parameterised entity $$\textsf{L}$$ specifies the country’s name, e.g. $$\texttt{UK}$$ and $$\texttt{China}$$. Entity $$\textsf{SCCs}$$ represents the Standard Contractual Clauses and is nested within $$\textsf{L}$$ entity to indicate the safeguards provided by $$\texttt{China}$$ are compliant^4^. $$\textsf{SCCs}$$ is also nested within $$\textsf{L}$$$$(\texttt{UK})$$ to allow us to check the safeguards as explained in Sect. 5.4. We present only one GDPR legal basis for cross-border data (providing a safeguard: SCCs). A method to model other location-based bases is provided in [27].

#### Example: agent, data types, and locations for the banking system

We show how these entities come together using the banking example, and the model initial state is in Fig. 3. The $$\textsf{Bank}$$ which itself is made of several components (a database and a notifier) is a data processor ($$\textsf{Proc}$$) as shown by the link into the $$\textsf{ADTypes}$$ perspective. Likewise the links denote $$\textsf{AdCompany}$$ as third party $$\textsf{TP}$$, and a $$\textsf{User}$$ as a data $$\textsf{Owner}$$. Specific components, e.g. $$\textsf{DB}$$, are created as nested components ($$\textsf{Comp}$$) inside $$\textsf{ADTypes}$$. Each $$\textsf{Comp}$$ links to: $$\textsf{AuthAgent}$$ if authorised; the permissions it can exercise on specific data; and the purposes of accessing that data. We specify the agent’s country by linking $$\textsf{Comp}$$ to its location in the $$\textsf{Locations}$$ perspective. For example, we link $$\textsf{AdCompany}$$ to $$\textsf{L}$$$$(\texttt{UK})$$.

The information being managed includes user $\textsf{Name}$ and transaction information ($\textsf{TransInfo}$) which are both assigned type $\textsf{Info}$ in $\textsf{ADTypes}$. The links to the $\textsf{DGE}$ specify how each type of $\textsf{Info}$ should be handled: $\textsf{Name}$ is $\textsf{PersonalInfo}$ and has store ($\textsf{S}$) permissions and $\textsf{Serving}$ purposes, while transaction information is general information (it is not linked to $\textsf{PersonalInfo}$) and allows read ($\textsf{R}$) access for $\textsf{Serving}$ and *optionally* can be used for advertising purposes.

The model is flexible, e.g. it is easy to remove $\textsf{AdCompany}$ (and associated links/entities in the privacy perspective) if this service is no longer being required.

### Consent perspective

As consent is shared between both the data owner and the $\textsf{DGE}$, we track this within an additional region (that each can reference). It is initially an empty perspective as consent is not specified in the model a-priori, but constructed during a model run using the rules presented in Sect. 5.

## Privacy dynamics

The static model describes the system setup, e.g. emphasising what agent is authorised and the permissions, but does not describe interaction with the system. To encode interaction we develop a set of privacy reaction rules. As with the static model, we give general purpose rules we expect to apply to a wide variety of examples, but bigraphs are open to extension and a modeller may add their own additional rules if required.

We categorise the rules into set for: (1) providing consent, (2) permission checking, (3) handling the right to access, (4) sharing data with a third party, (5) withdrawal of consent.

### Providing consent

**Fig. 4:**
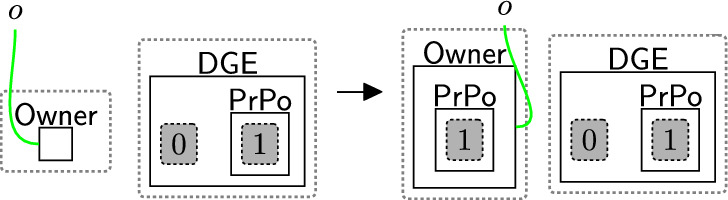
sendPolicy: sending privacy policies to the data owner

Before user data can be collected, their consent must be obtained. Rule sendPolicy (Fig. 4) models the sending of a policy from the $\textsf{DGE}$ to an owner. As we send policies to the abstract data owners, this same rule applies whether the owner is, for example, a banking customer or a patient in a healthcare system.

**Fig. 5:**
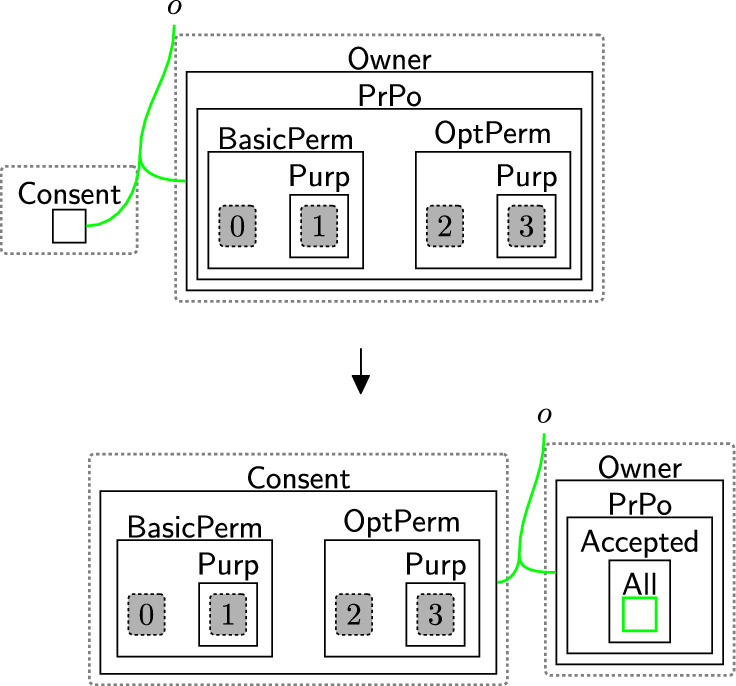
acceptAll: accepting all permissions and purposes

**Fig. 6:**
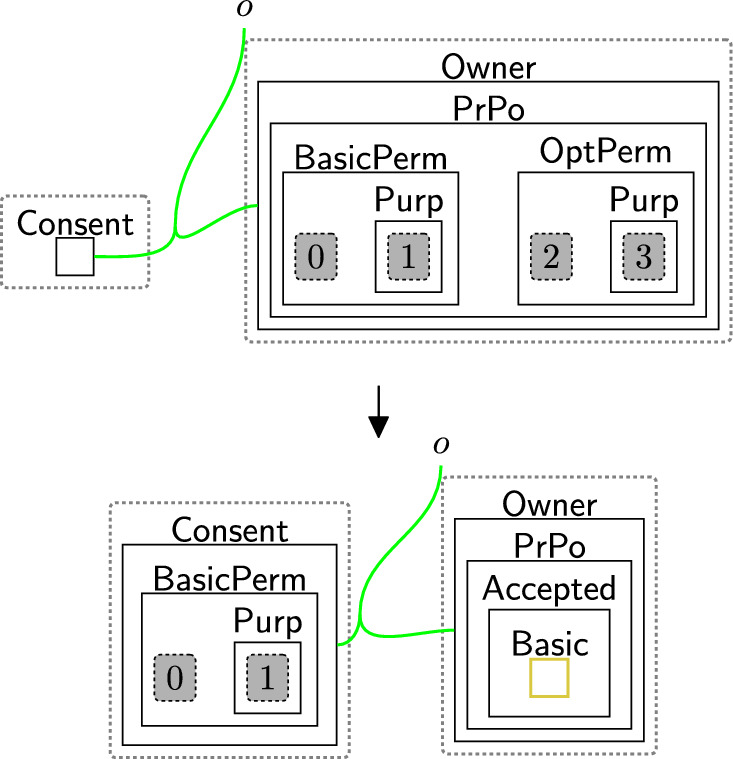
acceptBasic: accepting only basic permissions and purposes

**Fig. 7:**
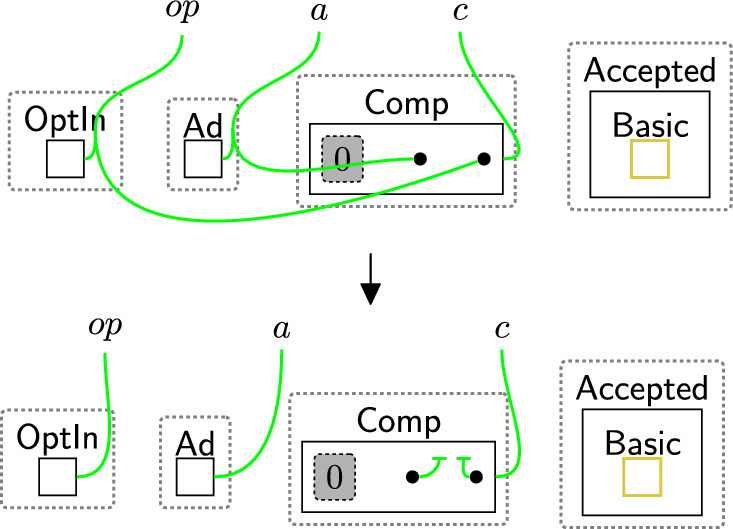
closeLinks: remove links between rejected permissions

**Fig. 8:**
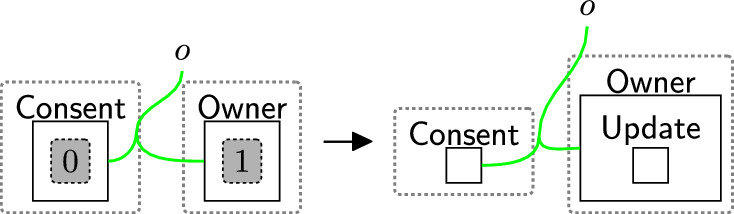
updateCons: owner’s request to update consent

**Fig. 9:**
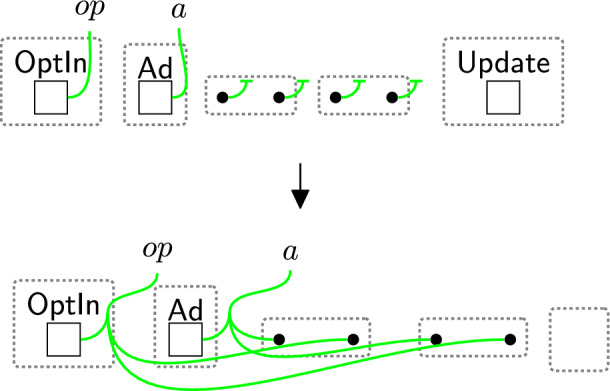
relinkPerm: relink the rejected permissions with their corresponding $\textsf{Comp}$ and $\textsf{Info}$

**Fig. 10:**
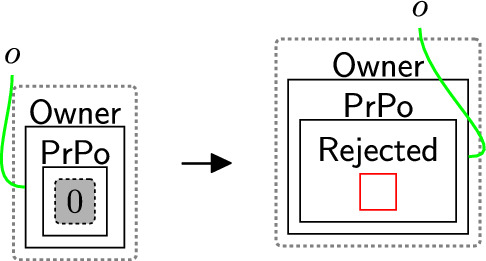
rejectAll: rejecting all permissions and purposes

Based on the policies, an owner can decide on what consent to give. The consented terms are placed in the $\textsf{Consent}$ perspective to explicitly express the notion of *freely given* and *genuine* consent [9, 40]. There are three cases:

1. Accept all $\textsf{BasicPerm}$, $\textsf{OptPerm}$, and $\textsf{Purp}$, as modelled by rule acceptAll (Fig. 5). Here, we track the owner has accepted all policies through a new entity $\textsf{All}$ and copy (through an instantiation map) the specific policies into the $\textsf{Consent}$ entity for that owner.
2. Accept only $\textsf{BasicPerm}$ and $\textsf{Purp}$ but reject optional permissions, as modelled by rule acceptBasic (Fig. 6). This is the case, for instance, when the data owner does not want to share their data with a third party. As before, we track the acceptance with a new entity $\textsf{Basic}$ and this time copy *only* the basic permissions and purposes to the $\textsf{Consent}$ entity for this user. A second set of rules (Fig. 7) closes links between the optional permissions in the $\textsf{DGE}$ perspective and the components of the third party in the $\textsf{ADTypes}$ perspective to indicate consent was rejected. This is a family of rules, and we define one per type of optional purposes, e.g. one for $\textsf{Ad}$, one for $\textsf{Marketing}$ etc. Because the framework is flexible, end-users can define a rule that allows data owners to selectively accept or reject individual optional permissions by tagging and recording the accepted ones in the consent perspective, while rejecting the others. Given that users may change their minds regarding their choices, we use rule updateCons (Fig. 8) to reset $\textsf{Consent}$ by removing its content and generate $\textsf{Update}$, which represents the $\mathsf {Owner's}$ request to update the consent. The entity $\textsf{Update}$ serves as a flag to apply rule $\textsf{relinkPerm}$ (Fig. 9), re-establishing the links for the rejected permissions. This, in turn, allows rule sendPolicy (Fig. 4) to be re-executed, enabling the user to revise their choices.
3. Reject all policies, as modelled by rule rejectAll (Fig. 10). We use the entity $\textsf{Rejected}$ to track the user’s rejection. As we do not have partial permissions like the reject optional case, we do not need to remove links as the privacy polices are never copied to the $\textsf{Consent}$ perspective^5^. In our model, the system treats the absence of consent the same as an explicit rejection since neither grants any permissions. Nevertheless, the end-user can verify if no consent is provided by checking for $\textsf{All}$, $\textsf{Basic}$ or $\textsf{Rejected}$ in the $\textsf{PrPo}$ that is nested within $\textsf{Owner}$. If neither entity is present, no consent is given.

**Fig. 11:**
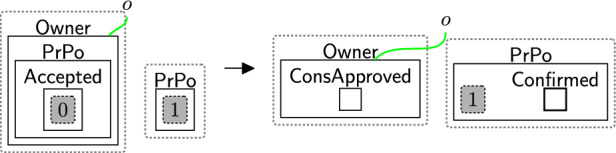
confirm: confirming a DGE has evidence of data owners consent

Once the owner has made their decision on the privacy policy their acceptance (either full or non-optional only) is confirmed by the DGE. This is needed as some privacy regulations, e.g. GDPR [41], require data controllers to keep evidence that proves the users consent. Confirmation is modelled through rule confirm (Fig. 11) that simply adds entities ($\textsf{Confirmed}$ and $\textsf{ConsApproved}$) to both the $\textsf{DGE}$ and owner. Site 0 represents the entity $\textsf{All}$ or $\textsf{Basic}$. It is deliberately not preserved in the right-hand side once the user’s decision is confirmed. Similarly, the owner’s copy of the privacy policy ($\textsf{PrPo}$) can be safely discarded, since the user’s choices are already recorded in the consent perspective.

### Permission checking

Before the system starts accessing (storing/reading) data the user’s consent must be checked to ensure they consented to these permissions and purposes.

As we set up links between the $\textsf{DGE}$, agents and data types, and consent perspectives based on the accepted polices, determining if a particular permission/purpose has been accepted is equivalent to checking a specific link exists. As we are interested in detecting incorrect accesses, we instead check for the absence of these links.

**Fig. 12:**
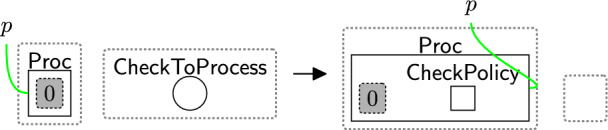
startCheck: the first rule that is used to start checking the permissions

**Fig. 13:**
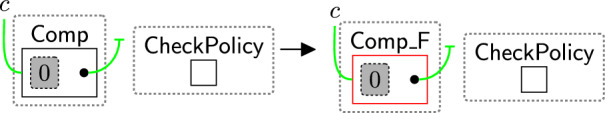
changeTypeComp: changing the component’s type that has a rejected permission/purpose

Checking the policies are valid follows a two-step process: (1) initialise the checking phase by using rule startCheck (Fig. 12), (2) iteratively check all links using rule change TypeComp (Fig. 13).

The entity $\textsf{CheckToProcess}$ (Fig. 12) should be generated by a system rule in the system perspective ($\textsf{Bank}$) to start the checking process. The privacy checking phase then uses rule startCheck (Fig. 12) to place a $\textsf{CheckPolicy}$ entity inside the $\textsf{Proc}$, indicating a policy check has started.

Once $\textsf{CheckPolicy}$ is generated, the checking process starts to check the absence of the links using rule change TypeComp (Fig. 13). This rule is used to change the type of any component that contains at least one permission/purpose with a closed link to $\mathsf {Comp\_F}$. For example, if the user rejected the optional permissions, the type of $\textsf{Comp}$ linked with $\textsf{Marketing}$ will be changed to $\mathsf {Comp\_F}$ to identify that $\textsf{Marketing}$ should be prevented from accessing the data.

We can check the policies multiple times, e.g. before executing each process, by allowing the system to generate the $\textsf{CheckToProcess}$ entity any time we need to check the consent. This handles, for example, policy updates over time.

### Right to access

**Fig. 14:**
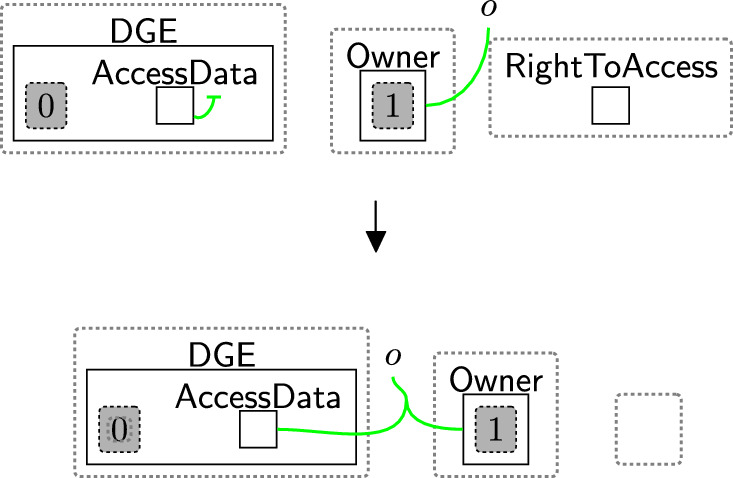
rightToAccess: allowing the owner to ask the $\textsf{DGE}$ for data access

Once the data is stored in the system, the user must be allowed to access their data at any time. The rule rightToAccess (Fig. 14) connects the owner to the $\textsf{DGE}$ (via the $\textsf{AccessData}$ entity) to indicate that the user can access the data. We will use this link to perform verification (Sect. 8).

Rule rightToAccess should be applied once the user’s data are stored in the system. To ensure that, when the checking process is done and the system stores the data, the entity $\textsf{CheckPolicy}$ should be replaced with $\textsf{RightToAccess}$ to allow this rule to be applied as it will be discussed in Sect. 6. After applying this rule, the entity $\textsf{RightToAccess}$ is deleted because there is no need to reuse the rule; the user can still access their data even if they update their permissions as permission updates do not affect the link between $\textsf{AccessData}$ and $\textsf{Owner}$.

### Sharing with third parties

Before sharing $\textsf{TransInfo}$ with the third party, we must check the user’s consent. If the type of $\textsf{Comp}$ linked to $\textsf{Marketing}$ is changed to $\mathsf {Comp\_F}$, sharing $\textsf{TransInfo}$ must be prevented, as the user has declined to share their information. Note: while $\textsf{TransInfo}$ may not always be considered personal data, it can often identify individuals (e.g. through payment history). Therefore, under regulations like the GDPR, it should be treated as personal data. Checking consent also ensures that the user’s data will be used in accordance with the purposes the user has accepted, in line with the purpose limitation principle

Some regulations, such as the GDPR, require safeguards like $\textsf{SCCs}$ for data transfers outside the EU. This means that before sharing data, we must determine whether the transfer is international (restricted) and, therefore, requires checking the safeguards. To do this, we first need to specify the locations of both the sender and the receiver. If they are in the same location, the data transfer can proceed. Otherwise, the transfer is restricted, and we must verify that the appropriate safeguards, such as $\textsf{SCCs}$, are in place.

**Fig. 15:**
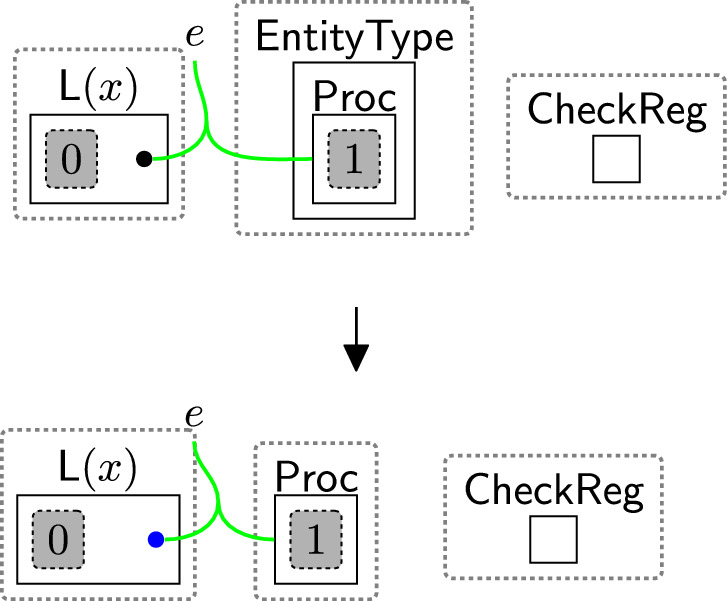
$\texttt {checkingReg}(x)$: checking the region of the sender/receiver by tagging their pointers, where *x* is the name of a country or a jurisdiction

**Fig. 16:**
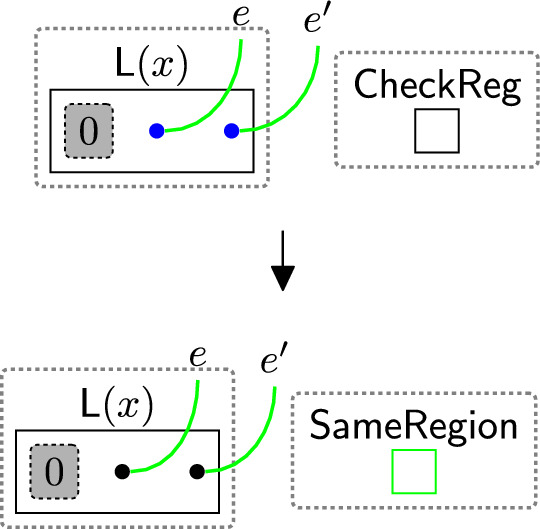
$\texttt {sameRegion}(x)$: the sender and the receiver are in the same location. *x* is the name of a country or a jurisdiction

**Fig. 17:**
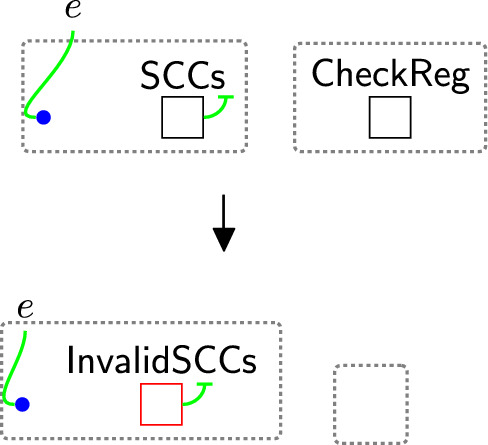
changeTypeSCCs: changing the type of the entity SCCs that has a closed link

Rule $\texttt {checkingReg}(x)$ (Fig. 15) determines the locations of the sender/receiver by tagging the pointers linked to their types in the $\textsf{ADTypes}$ perspective. The parameter *x* is the name of a country or a jurisdiction, e.g. the $\texttt{EU}$. $\textsf{EntityType}$ specifies the sender’s/receiver’s type, e.g. $\textsf{Proc}$, $\textsf{TP}$, etc. $\textsf{CheckReg}$ initiates the region-checking process. These entities are generated by system rules as we will discuss in Sect. 6 to trigger the subsequent rules sameRegion and changeTypeSCCs (Figs. 16, 17) as explained further below in this section.

Suppose the user consents to share their data with $\textsf{AdCompany}$ and its branch $\textsf{Marketing}$. Before sharing, we must check if the transfer is restricted. We use rule $\texttt {checkingReg}(x)$ (Fig. 15) to tag the pointer linked to the sender’s type ($\textsf{Proc}$, since the $\textsf{Bank}$ is the processor). The same rule is applied to tag the pointers linked to the receiver types, replacing $\textsf{Proc}$ with $\textsf{TP}$ for $\textsf{AdCompany}$ and $\textsf{Comp}$ for $\textsf{Marketing}$ (see Figs. 54 and 53).

If the tagged pointers are located in the same regions, rule $\texttt {sameReg}(x)$ in Fig. 16 is applied. The rule replaces the entity $\textsf{CheckReg}$ with $\textsf{SameRegion}$ to indicate that the sender and receiver are in the same region, allowing the data to be safely shared without checking the $\textsf{SCCs}$. We then untag the pointers so that the rule can be reused if needed.

If rule $\texttt {sameReg}(x)$(Fig. 16) is not matched, it indicates that the sender and receiver are in different countries. In this case, we must verify the safeguards ($\textsf{SCCs}$).

Our objective is to identify invalid $\textsf{SCCs}$. If the $\textsf{SCCs}$ nested within the sender country are linked to the $\textsf{SCCs}$ in the recipient country, then the data can be safely transferred. Otherwise, the $\textsf{SCCs}$ are considered invalid. For example, the $\textsf{SCCs}$ nested within the sender country ($\texttt{UK}$) (Fig. 3) is not linked to the $\textsf{SCCs}$ in the recipient country ($\texttt{China}$); then, rule changeTypeSCCs (Fig. 17) changes the type of $\textsf{SCCs}$ to $\textsf{InvalidSCCs}$. This change indicates that the $\textsf{SCCs}$ are invalid, e.g. not accepted by the sender, so the transfer should be prevented. We can then omit the $\textsf{CheckReg}$ entity to terminate the checking process.

### Withdrawing consent

Just as users have the right to provide consent and access their data, they also have the right to withdraw their consent at any time. Users can withdraw both $\textsf{OptPerm}$ and $\textsf{BasicPerm}$ or choose to withdraw only the $\textsf{OptPerm}$. Importantly, the rules governing consent withdrawal must be applicable at any point during the system’s state transitions. This is achieved through the use of priority classes, enabling users to revoke their consent—either fully or partially at any stage of the system’s evolution.

#### Withdrawing all permissions

**Fig. 18:**
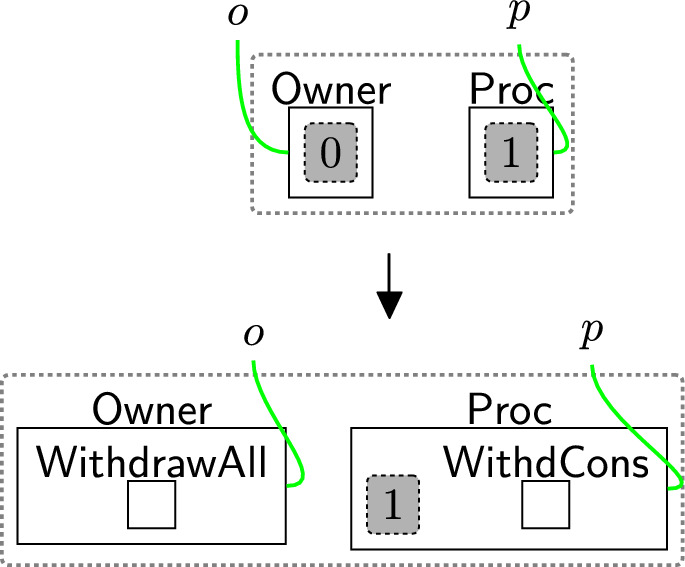
withdrawReq: the owner requests to withdraw all permissions

Withdrawing consent is a three-step process (and uses three rules). First, a user sends a consent withdraw request through rule withdrawReq (Fig. 18). This places the entity $\textsf{WithdCons}$ in the $\textsf{Proc}$. There is no need to specify which user should withdraw their consent as the model supports one user.

**Fig. 19:**
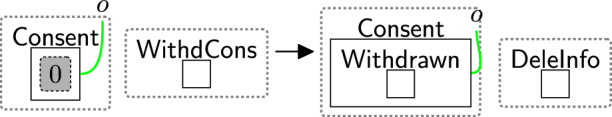
processWithdrawal: the consent is withdrawn and the user’s data should be deleted from the system

The processor then actions the withdrawal using reaction rule processWithdrawal (Fig. 19) that removes all permissions from the consent perspective, by removing the site and replacing it with a $\textsf{Withdrawn}$ entity, and discarding $\textsf{WithdCons}$ as the process of withdrawing the consent is done. $\mathsf {{DeleInfo}}$ is used to delete existing data. Rule(s) to delete data are system-specific as they need information, for example, database models as discussed in Section 6. The deletion process does not necessarily occur immediately after the withdrawal process e.g. to allow companies to use batched deletions.

**Fig. 20:**
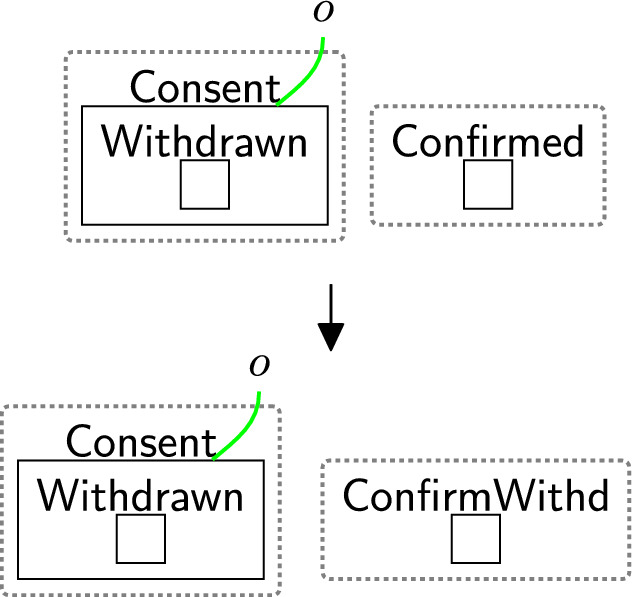
confirmWithdrawal: acknowledgement of successful consent withdrawal

Finally, we confirm that consent is withdrawn using reaction rule confirmWithdrawal (Fig. 20). This step is not a specific requirement of the regulations, but is used to replace the confirmation of consent with the withdrawal. In this case, we simply change $\textsf{Confirmed}$ to $\textsf{ConfirmWithd}$ to record this operation.

**Fig. 21:**
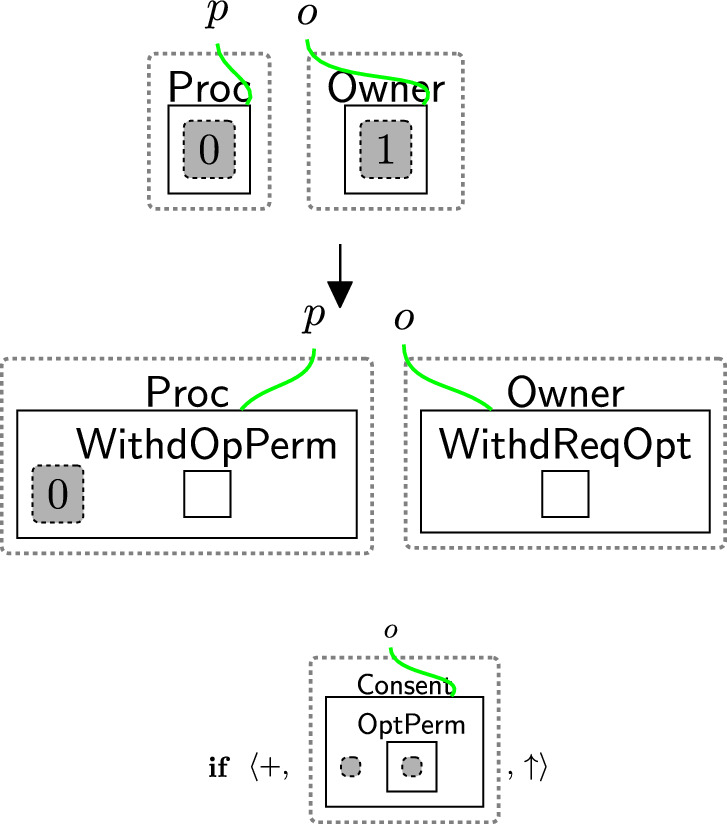
withdrawReqOPT: the owner requests to withdraw the optional permissions. The condition allows the rule to be applied only if the owner has already accepted the optional permissions

**Fig. 22:**
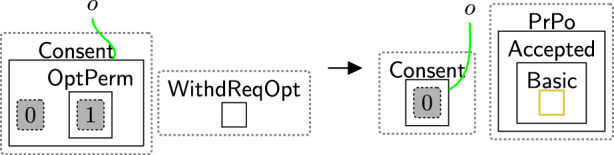
processWithdOpt:the optional permissions are withdrawn and regenerate $\textsf{Basic}$ to recheck the consent

#### Withdrawing only optional permissions

Similar to withdrawing all the permissions, we have three steps to process withdrawing $\textsf{OptPerm}$. Rule withdrawReq OPT (Fig. 21) models the owner’s request to withdraw the optional permission. This rule adds the entities $\textsf{WithdReqOpt}$ in $\textsf{Owner}$ and $\textsf{WithdOpPerm}$ in $\textsf{Proc}$ to allow the processor to start processing the partial withdrawal. The condition restricts the rule’s application as the rule should be applied only if the user accepted all the permissions.

Rule processWithdOpt in Fig. 22 allows the user to update the consent by accepting only the $\textsf{BasicPerm}$ and deleting $\textsf{OptPerm}$ from the $\textsf{Consent}$ perspective.

As the consent is updated, we must close the links of the rejected permissions and recheck the consent by generating the entity $\textsf{CheckToProcess}$ as discussed in Sect. 6.

## Integrating privacy and system-specific behaviour

As shown in Fig. 1, a model consists of a reusable set of privacy entities/rules coupled to a system-specific model. We describe how the system model interacts with the privacy model by showing some of the rules needed in the banking example. We assume GDPR requirements and that the user has accepted the basic permissions required for interacting with the system.^6^

**Fig. 23:**
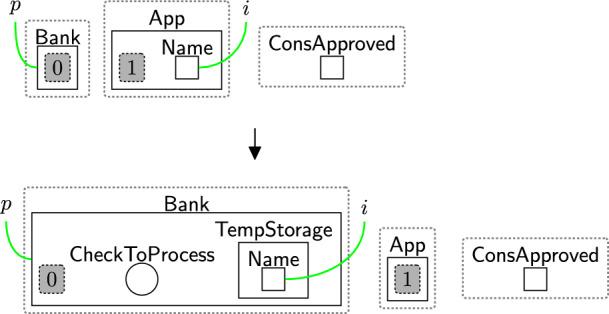
registerRequest: user’s registration with the system

Once the consent is approved (using privacy rules), the system can register a user by sending their $\textsf{Name}$ to the $\textsf{Bank}$ as shown in Fig. 23. The rule explicitly matches over $\textsf{ConsApproved}$ that is generated by rule confirm (Fig. 11) to show a user has accepted some policies (but not the specific policies). Before starting to store or process user data, we must check the user’s consent.

To do so, the user’s $\textsf{Name}$ is first temporarily stored in $\textsf{TempStorage}$ before being stored in the database. The entity $\textsf{CheckToProcess}$ is generated to tell the *privacy model* to begin the checking process by enabling rule startCheck (Fig. 12), i.e. $\textsf{CheckToProcess}$ is a special flag that allows integration between the models.

**Fig. 24:**
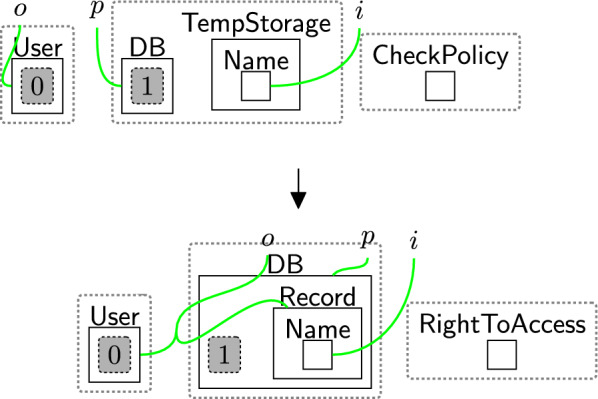
storingName: storing the user’s name

After checking, the system continues based on the checking result. As the user accepted the basic permissions, the bank is now able to store the user’s name using rule storingName (Fig. 24). To maintain provenance of the data, we link the $\textsf{User}$ with the $\textsf{Record}$ on link *o*. For the GDPR and other regulations with a notion of right to access, this rule must be followed by rule rightToAccess (Fig. 14), as the user must be allowed to access their data. To do that, the entity $\textsf{RightToAccess}$ should be generated as shown in Fig. 24 to allow rule rightToAccess (Fig. 14) to be applied directly after storing the data.

**Fig. 25:**
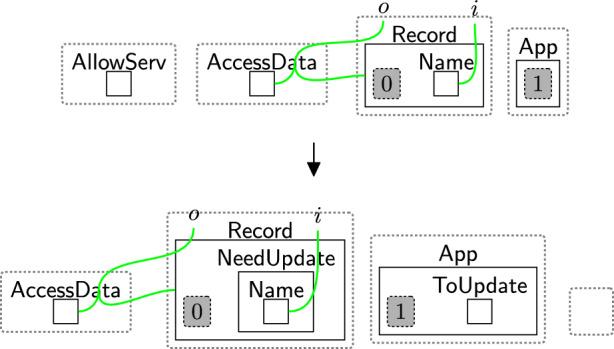
updateReq: user’s request to update their data

The user now may ask to access their data. For example, rule updateReq (Fig. 25) models their request to retrieve and update their record. Here, $\textsf{ToUpdate}$ denotes the request and $\textsf{NeedUpdate}$ specifies which information must be changed. We omit $\textsf{AllowServ}$ once processing begins and regenerate it upon completion, allowing the user to make further requests. The entity $\textsf{AllowServ}$ is generated by the system (see Fig. 44 Appendix A). By explicitly matching on $\textsf{AccessData}$ (after linking it to $\textsf{Owner}$), we can ensure that the user can always access their data.

### Sharing data based on consent

**Fig. 26:**
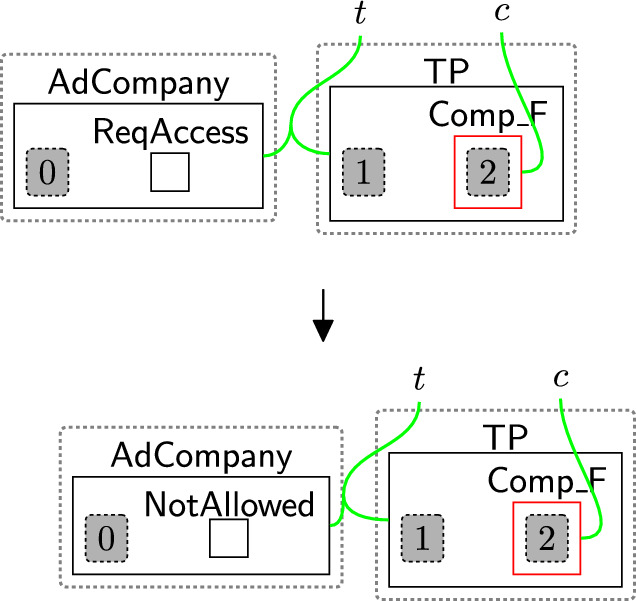
preventAdCompany: prevent sharing the user’s transaction information with $\textsf{AdCompany}$ as the user rejects the optional permissions

**Fig. 27:**
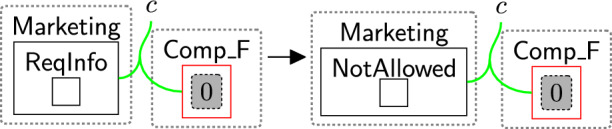
preventMarBraCons: prevent sharing the user’s data with the marketing department

Since the consent is checked before processing the user’s data (as mentioned earlier), the $\textsf{Comp}$ linked to $\textsf{Marketing}$ is changed to $\mathsf {Comp\_F}$ because the user has only accepted basic permissions. Rule preventAdCompany (Fig. 26) and preventMarBraCons (Fig. 27) are then applied to prevent information sharing with the marketing branch and its parent company ($\textsf{AdCompany}$).

If there were no components with type $\mathsf {Comp\_F}$, then the user accepted the optional permissions, so we should start checking the second requirement.

**Fig. 28:**
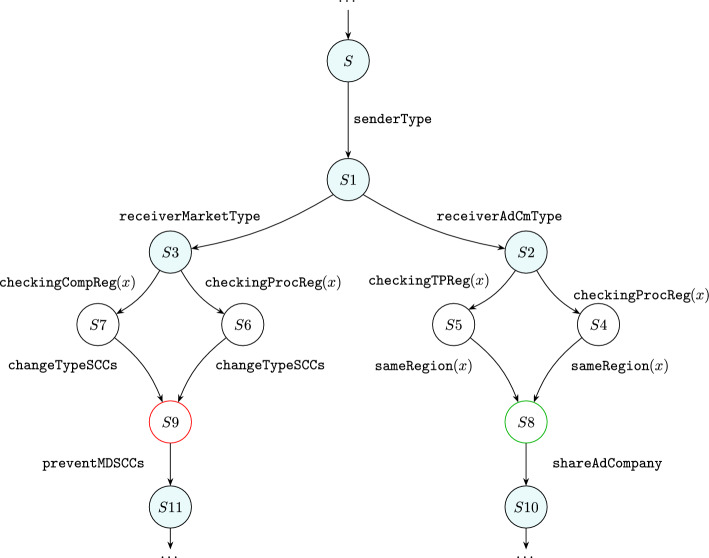
Partial transition system illustrating the interaction between system and privacy rules. Teal states are produced by applying a system rule

### Transferring data based on GDPR requirements for international transfer

If the user accepts the optional permissions, we need to determine whether the data transfer is international before proceeding. If so, we must check the GDPR requirements for cross-border data transfers.

To check if the transfer is international, we need to specify the sender’s and receiver’s locations using rule $\texttt {checkingReg}(x)$ (Fig. 15). However, to be able to use the rule, we must first define systems rules that specifies the sender’s and receiver’s types. Figure 28 shows the interaction between the system and privacy rules, where teal states are produced by system rules and others by privacy rules.

**Fig. 29:**
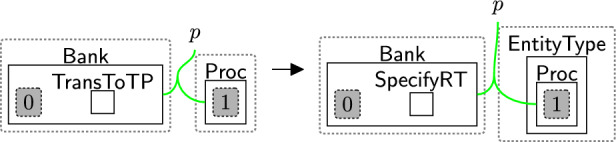
senderType: specifying the type of $\textsf{Bank}$

For example, state *S*1 is produced by applying rule senderType (Fig. 29), a system rule defining the sender’s type ($\textsf{Bank}$). This rule generates $\textsf{EntityType}$ around $\textsf{Proc}$, indicating that the $\textsf{Bank}$ is a processor. $\textsf{TransToTP}$ is generated by another system rule (see Figs. 48, 49) to indicate that the system will transfer the data to a third party. It is replaced with $\textsf{SpecifyRT}$ to start determining the type of the receiver(s). $\textsf{SpecifyRT}$ is nested within the $\textsf{Bank}$ because the $\textsf{Bank}$, as the sender, is responsible for specifying the receiver’s type.

**Fig. 30:**
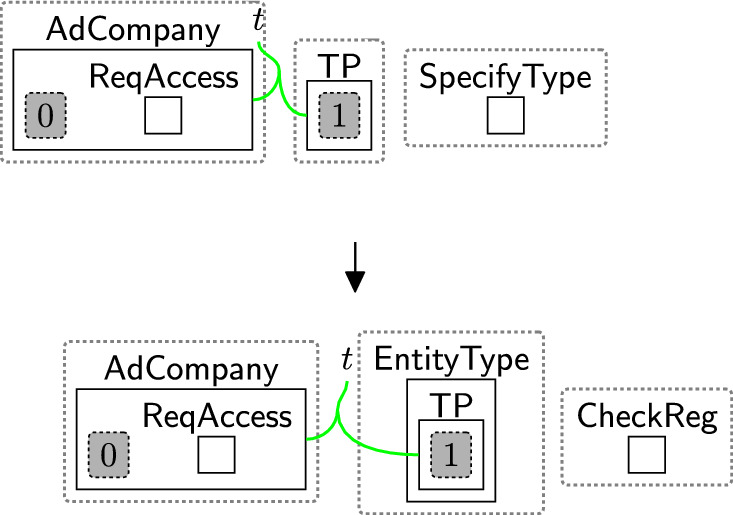
receiverAdCmType: specifying the type of $\textsf{AdCompany}$

We have two receivers in this example: $\textsf{AdCompany}$ and $\textsf{Marketing}$. To specify the type of $\textsf{AdCompany}$, we use rule receiverAdCmType (Fig. 30). As $\textsf{AdCompany}$ is a third party, the entity $\textsf{EntityType}$ is generated around the $\textsf{TP}$. We replace the entity $\textsf{SpecifyRT}$ with $\textsf{CheckReg}$. After applying this rule, state *S*2 is produced as shown in Fig. 28.

**Fig. 31:**
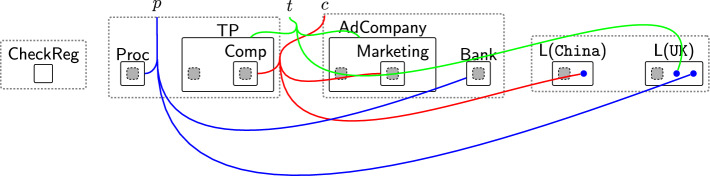
The tagged pointers after specifying the location of $\textsf{Bank}$, $\textsf{AdCompany}$, and $\textsf{Marketing}$. The tagged pointers of $\textsf{Bank}$ and $\textsf{AdCompany}$ are nested within $\texttt{UK}$, while the tagged pointer of $\textsf{Marketing}$ is in $\texttt{China}$

**Fig. 32:**
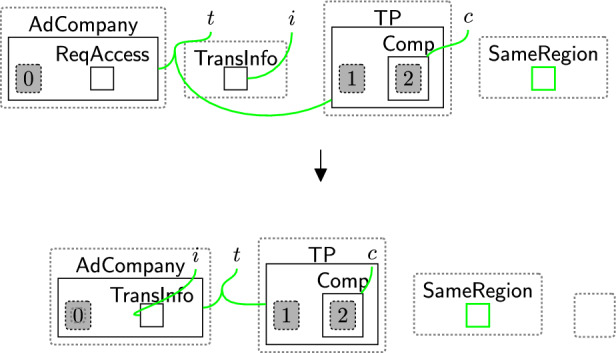
receiverMarketType: specifying the type of $\textsf{Marketing}$

As we generate the entity $\textsf{CheckReg}$, rule $\texttt {checkingReg}(x)$ (Fig. 15) is triggered to tag the pointer liked to the $\textsf{Bank}$ (*S*4). We use the same rule to tag the pointer linked to $\textsf{AdCompany}$, but we should replace $\textsf{Proc}$ with $\textsf{TP}$ (*S*5). As shown in Fig. 31, both tagged pointers are located in the $\texttt{UK}$, meaning that the sender ($\textsf{Bank}$) and receiver ($\textsf{AdCompany}$) are both in the $\texttt{UK}$. Thus, rule $\texttt {sameReg}(x)$ (Fig. 16) is applied (*S*8). Now, we can share the data without checking the $\textsf{SCCs}$ by applying rule Fig. 32 (*S*10). In this rule, we match on $\textsf{Comp}$ and $\textsf{SameRegion}$ to ensure the sharing process is performed after checking the requirements (checking consent and the requirements of the international data transfers).

**Fig. 33:**
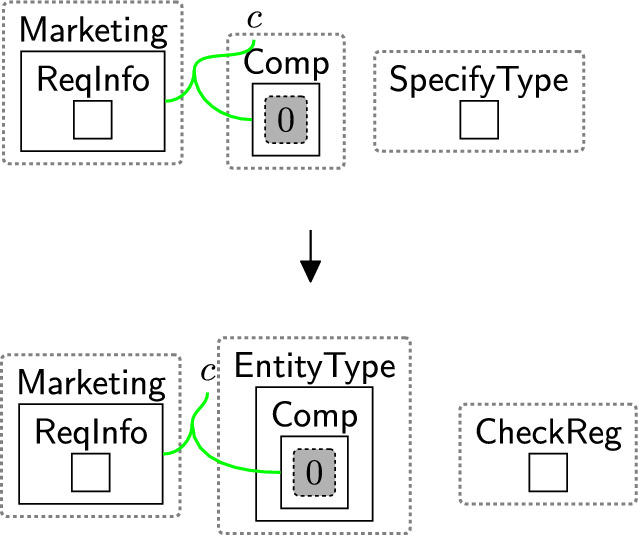
shareAdCompany: sharing the data with $\textsf{AdCompany}$

**Fig. 34:**
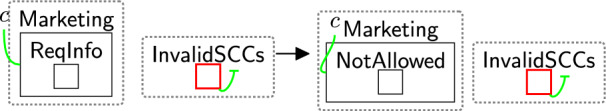
preventMDSCCs: prevent sharing the user’s transaction information with $\textsf{Marketing}$ as the used $\textsf{SCCs}$ is invalid

Similarly, we use rule receiverMarketType (Fig. 33) to specify the type of $\textsf{Marketing}$ (*S*3). We also use rule $\texttt {checkingReg}(x)$ (Fig. 15) to tag the pointer liked to the $\textsf{Bank}$ and the pointer linked to $\textsf{Marketing}$ (*S*6 and *S*7, respectively). As shown in Fig. 31, the tagged pointer linked to $\textsf{Marketing}$ is in $\texttt{China}$, while the pointer of $\textsf{Bank}$ is located in the $\texttt{UK}$. In such a case, rule changeTypeSCCs (Fig. 17) is applied to change $\textsf{SCCs}$ to $\textsf{InvalidSCCs}$ (*S*9) because the $\textsf{SCCs}$ nested within the $\texttt{UK}$ and the $\textsf{SCCs}$ nested within $\texttt{China}$ are not linked. This means we must not transfer the data to $\textsf{Marketing}$ even if the user **consented** to do so as it does not meet the GDPR requirements for international data transfer. Rule preventMDSCCs(Fig. 34) prevents sharing the data with $\textsf{Marketing}$ (*S*11).

### Withdrawing consent

**Fig. 35:**
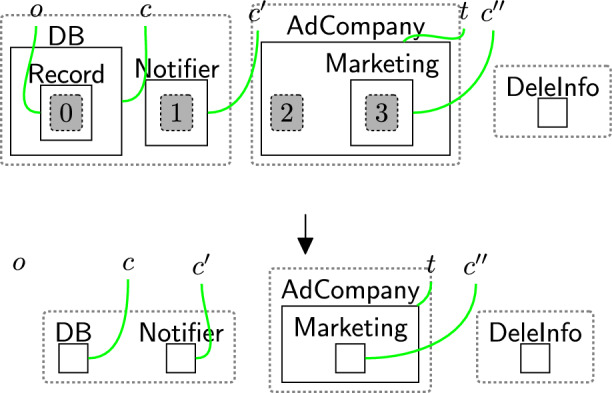
deleteInfo: deleting the user’s information after withdrawing the consent

The user also should be able to withdraw the consent at any time, and the consent should be withdrawn once the user asks for that (rule withdrawReq in Fig. 18 and processWithdrawal in Fig. 19). After withdrawing the consent, the rule deleteInfo (Fig. 35) deletes the user’s information from $\textsf{DB}$, $\textsf{Notifier}$, $\textsf{AdCompany}$, and $\textsf{Marketing}$. To ensure that rule deleteInfo (Fig. 35) is applied after withdrawing the consent, we explicitly match on $\textsf{DeleInfo}$ that is generated by rule processWithdrawal in Fig. 19. The last step is confirming the withdrawal by using rule confirmWithdrawal (Fig. 20).

**Fig. 36:**
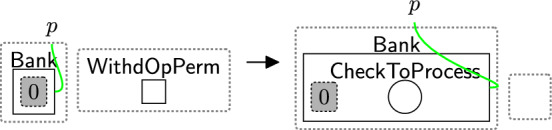
recheckCon: initialising the process of rechecking consent after withdrawing optional permissions

The user also has the right to withdraw only the $\textsf{OptPerm}$. Rule withdrawReqOPT (Fig. 21) and processWithdOpt (Fig. 22) are applied to perform the withdrawal step of the optional permissions. After performing the partial withdrawal, we use rule closeLinks (Fig. 7) to close the links of the rejected permissions. Because the consent is updated, we should recheck it before starting processing the user’s data. Rule recheckCon (Fig. 36) generates the flag $\textsf{CheckToProcess}$ to trigger rule startCheck in Fig. 12 to start checking the consent. We match on $\textsf{WithdOpPerm}$ (generated by rule withdrawReqOPT in Fig. 21) to allow rule startCheck (Fig. 12) to be applied after withdrawing the optional permissions.

**Fig. 37:**
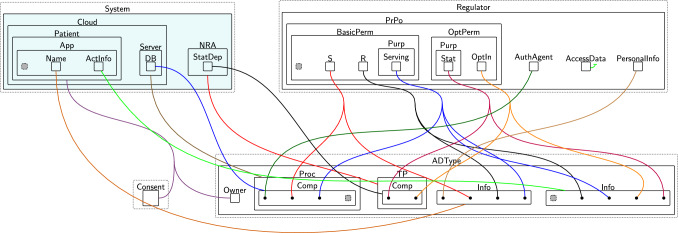
A (partial) initial state of the cloud-based home healthcare system

## Reusability: modelling a home healthcare system

To demonstrate our approach is reusable and applicable to a variety of examples and regulations, we model a second example: a cloud-based home healthcare system. Instead of GDPR, we apply the California Consumers Privacy Act (CCPA). The privacy aspects of this example are derived from the privacy policies of the Fitbit app for users in California [42]. Here, a patient uses Fitbit app that records their physical activity level ($\textsf{ActInfo}$). The data is stored on, and advice is generated on, a cloud server. Patient information can be shared with a third party to inform studies in population health [43]: the statistics department ($\textsf{StatDep}$) at the National Regional Authority ($\textsf{NRA}$).

Modelling this system requires creating new system-specific entities^7^, e.g. $\textsf{NRA}$, $\textsf{Server}$, $\textsf{Patient}$, linking them to the appropriate agent and data types, and specifying new privacy policies for the $\textsf{DGE}$. An example (partial) initial state is in Fig. 37 where we use colours to differentiate the types of linking.

The $\textsf{Server}$ is the data processor in this system, and $\textsf{DB}$ is its component. The third party is $\textsf{NRA}$ and $\textsf{statDep}$ is its agent. $\textsf{DB}$ is an authorised agent, while $\textsf{statDep}$ is unauthorised. The $\textsf{DB}$ can store the $\textsf{Patient}$’s $\textsf{Name}$ and $\textsf{ActInfo}$.

End-users can amend the privacy framework to model the CCPA by removing unnecessary privacy entities. For example, since the CCPA does not specify requirements for cross-border data transfers, there is no need to use the $\textsf{Locations}$ perspective, as shown in Fig. 37.

Alongside defining the initial state, they should use the privacy rules outlined in Sect. 5. To adapt the framework to their needs, the end-users can use only the privacy rules they require, discard the others, or adjust the priority classes of these rules. To illustrate this, the CCPA has the notion of *notice at collection*, which assumes users agree to the privacy policy by default, but they are able to request that their data not be shared at any time^8^. To model this notion, we use rule sendPolicy (Fig. 4) once the user asks to use the system. Rule acceptAll (Fig. 5) is used by default, e.g. instantaneous rule, a higher priority rule, allowing it to be applied immediately and invisibly within the system. Rule acceptBasic (Fig. 6) and confirm (Fig. 11) can be discarded since the users cannot choose the policy they want to consent to, and consent confirmation is not a CCPA requirement.

Like the GDPR, the CCPA allows users to access their data at any time. After storing the patient’s name, we use rule rightToAccess (Fig. 14) to explicitly match over $\textsf{AccessData}$ in each system rule that represents a user’s request to access their record as discussed in Sect. 6.

**Fig. 38:**
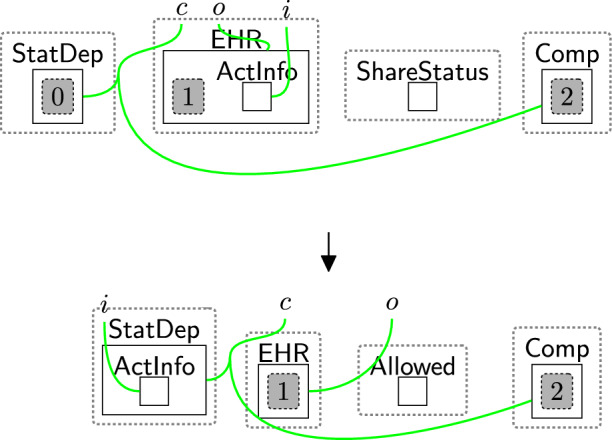
shareActInfo: sharing the patient’s activities information with the statistical department at the NRA

When the patient starts generating activities, the recorded information about their activities ($\textsf{ActInfo}$) is shared with $\textsf{StatDep}$ if the user did not ask to stop sharing their data. Rule shareActInfo (Fig. 38) shares $\textsf{ActInfo}$ with $\textsf{StatDep}$. It matches over $\textsf{Comp}$ to ensure that the $\textsf{Patient}$ did not ask data sharing to end.

**Fig. 39:**
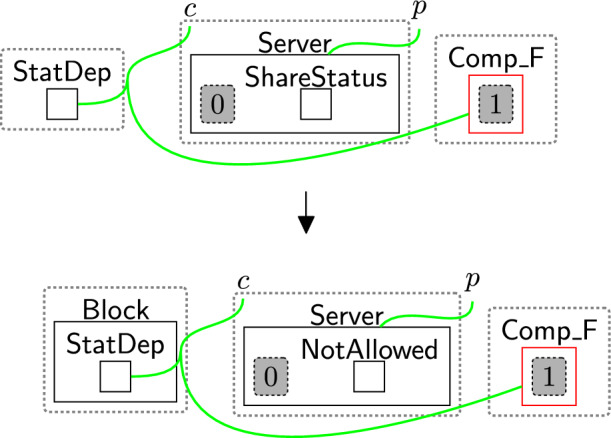
preventshareActInfo: prevent sharing the patient’s activities information with the statistical department at the NRA

Once the user asks to stop sharing their data, rules withdrawReqOPT (Fig. 21) and processWithdOpt (Fig. 22) should be applied. We also should use rule closeLinks (Fig. 7) to terminate the sharing permissions. The consent in this case should be rechecked to change the components’ types of the third party to $\mathsf {Comp\_F}$. We define a system rule shown in Fig. 39 that explicitly matches over $\mathsf {Comp\_F}$ to prevent sharing $\textsf{ActInfo}$ with $\textsf{StatDep}$.

We can also use rule updateCons (Fig. 8) and rule relinkPerm (Fig. 9) to enable users to update their decisions after withdrawing optional permissions. In this case, we need to reset the type of $\mathsf {Comp\_F}$ to $\textsf{Comp}$.

**Fig. 40:**
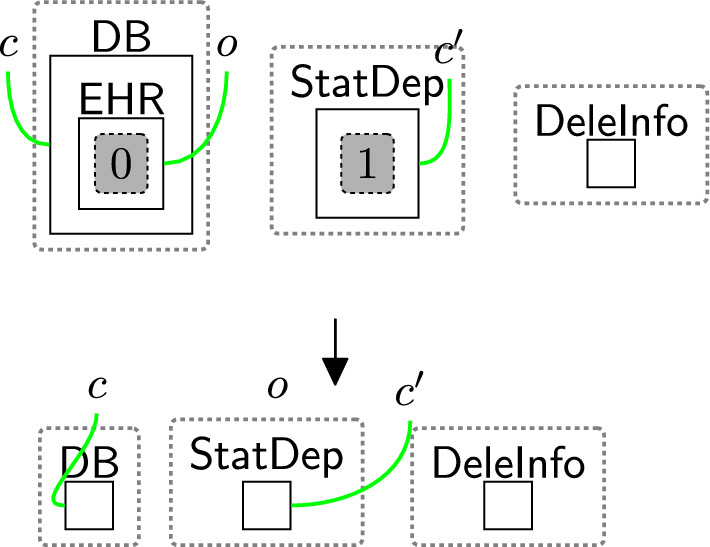
deleteInfoEHR: deleting the patient’s information after withdrawing the consent

To model the notion of *right to delete* [6], we use rule withdrawReq (Fig. 18) and processWithdrawal (Fig. 19). After applying these two rules, the entity $\textsf{DeleInfo}$ is generated, which we use to define a system rule that deletes the $\textsf{Patient}$’s electronic health record ($\textsf{EHR}$) as shown in Fig. 40.

## Verification

We perform two types of analysis: (1) model checking that considers a *specific* system’s configuration and determines if there can be potential breaches of privacy regulations in future, and (2) static analysis, where we focus on the structure of the rules to show certain states can never exist (in any model).

### Model checking

Once we have a formal model of privacy we can utilise it to perform *model-checking verification*: checking the system, and its privacy policies, provably adheres to a specific regulation.

First, we create a transition system representing all possible updates the system can make. Given an initial state of the system, the transition system is *automatically* generated using BigraphER, with bigraphs representing states, and reaction rule applications representing transitions.

Manually inspecting the resulting transition system to prove privacy properties in our banking and healthcare examples is challenging due to the large state spaces: with 396 states in the banking example and 182 states in the healthcare example. To manage this complexity, we use the PRISM [18] model checker as it is supported natively by BigraphER.

Although PRISM supports probabilistic properties, our privacy specifications—based on the regulations—are written in the non-probabilistic fragment of PRISM’s property specification language which subsumes logics like CTL [45]. We use the CTL-like elements and introduce the syntax/semantics as they are used.

Finally, to allow labelling of states, used within the logical specifications, *bigraph patterns* (that act like left-hand side matches in rewriting) are added to our model. Often these are used to simply check for the presence of a specific entity in a state. We can also use them to detect design flaws by searching for specific conditions within the rules. For space, where the predicates are trivial we show only the predicates for the first property, but the rest are available in the full model file [30]. Importantly, privacy predicates are predefined, i.e. they represent bigraph patterns of the right-hand side of the privacy rules. However, adjustments are needed for the predicates related to the system’s perspective, which are highlighted in teal in this section.

**Fig. 41:**
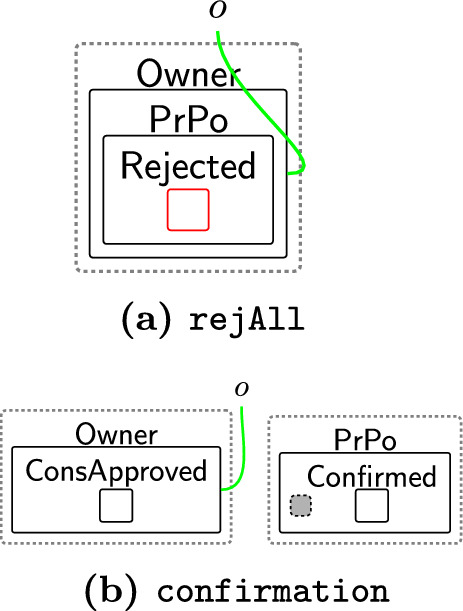
Example bigraph patterns

We first check a system does not assume consent unless the user explicitly accepts the policies. In this case, it is easier to verify the inverse statement: consent is never confirmed if a user rejects the policies. We use the following CTL formula:

1 $$\begin{aligned} \textbf{A}\left[ \, \textbf{G}\, ( \texttt{rejAll} \implies \lnot \texttt{confirmation} )\,\right] \end{aligned}$$

where $\texttt{rejAll}$ and $\texttt{confirmation}$ are bigraph predicates that label states whenever the bigraphs shown in Fig. 41a, b are matched. $\textbf{A}$ is a path quantifier meaning *for all possible (future) paths*, while $\textbf{G}$ is *globally*, i.e. the property must hold for all future states on the path.

A related property is that we must ensure consent is given before we start any data processing. Expressed in CTL:

2 $$\begin{aligned} \qquad \qquad \textbf{A} \left[ \, (\lnot \texttt{process})\, \textbf{W}\, \texttt{checkingCons} \, \right] \end{aligned}$$

Here we use $\textbf{W}$ which is the *weak until* operator. In our case, this means that $\texttt{process}$ (store/copy etc.) should never ($\lnot$) hold until after $\texttt{checkingCons}$ (consent has been checked) holds. The weakness means that $\texttt{checkingCons}$ does not need to hold at any point, e.g. if the user rejects the policies, then it is still true that there was no processing done before consent was checked.

We also ensure that the system does not share the user’s data if the user withdraws the optional permissions using the following formula:

3 $$\begin{aligned} \textbf{A} \left[ \, \textbf{G}\, ( \texttt{partWithd} \implies (\textbf{X} \lnot \texttt{shareInfo} )) \, \right] \end{aligned}$$

This property ensures that for all paths, if the user withdraws optional permissions ($\texttt{partWithd}$), then in the very next state ($\textbf{X}$), data is not shared with third parties ($\lnot$ $\texttt{shareInfo}$) as soon as the permission is withdrawn. The aim of using next operator $\textbf{X}$ is to ensure that once the permissions are withdrawn, the system instantly ceases any sharing in the very next state.

Similar to property (3), we check that optional processing is handled correctly and that the user’s data is not shared if the $\textsf{SCCs}$ are invalid:

4 $$\begin{aligned}  &   \textbf{A} \left[ \textbf{G} (\texttt{rejectOpt} \implies (\textbf{X} \lnot \texttt{shareInfo})) \right] \end{aligned}$$

5 $$\begin{aligned}  &   \textbf{A} \left[ \, \textbf{G}( \texttt{invalidSCCs} \implies (\textbf{X} \lnot \texttt{shareInfo} )) \, \right] \end{aligned}$$

We check that users who store information always have the right to access it by using the following formula:

6 $$\begin{aligned} \textbf{A} \left[ \, \textbf{G}\, ( \texttt{storeInfo} \implies (\textbf{F}\, \texttt{accessRight}) )\, \right] \end{aligned}$$

This formula uses an additional operator $\textbf{F}$ that denotes *eventually*. This means $\texttt{accessRight}$ does not need to immediately hold, i.e. the action that stores the info ($\texttt{storeInfo}$) fires at some point and then there may be multiple steps before the access becomes available e.g. to create the access link, but it always will.

We can verify there is never unauthorised access, e.g. the links never connect where they should not or personal information is not nested within an unauthorised agent:

7 $$\begin{aligned} \textbf{A} \left[ \, \textbf{G}\, \lnot \texttt{unauthAccess} \,\right] \end{aligned}$$

Here, the entire property is inverted, i.e. for all paths there is no an unauthorised access.

Finally, we check that the user’s data is deleted when the consent is withdrawn:

8 $$\begin{aligned} \textbf{A} \left[ \, \textbf{G}\, ( \texttt{withdAll} \implies (\textbf{F}\, \texttt{deletingInfo}) )\, \right] \end{aligned}$$

With this formula, we ensure that in every possible execution of the system, if consent is fully withdrawn at any point, the system will eventually delete the user’s information, although the deletion may occur after several steps following the withdrawal of consent.

All these properties hold for both our banking and healthcare examples when checked with PRISM. This gives increased confidence^9^ the systems, and privacy policy implementations, operate in accordance with the privacy regulations: (1) and (5) say we are GPDR compliant, while the remaining properties are shared by all the regulations we have considered.

### Detecting privacy violations

**Fig. 42:**
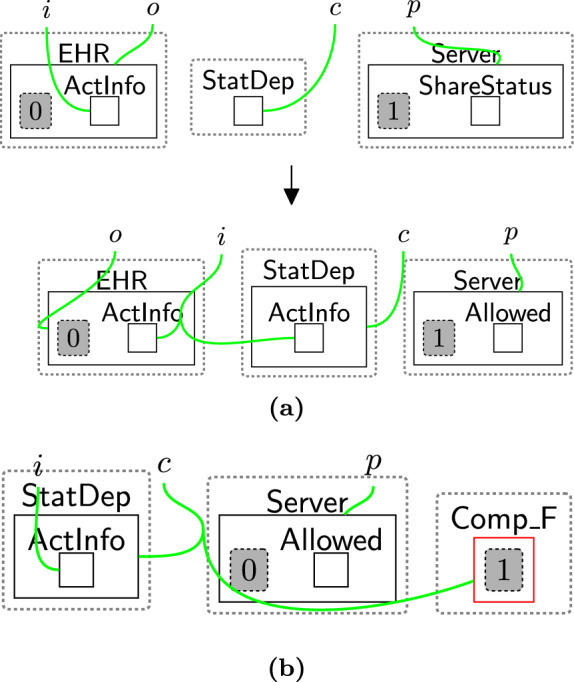
**a** System-specific rule for copying data to a third party. As the consent was not checked (i.e. there is no match into the privacy perspectives), this may result in a privacy violation; **b** partial model state showing the privacy violation: activity information has moved when consent was not given

**Fig. 44:**
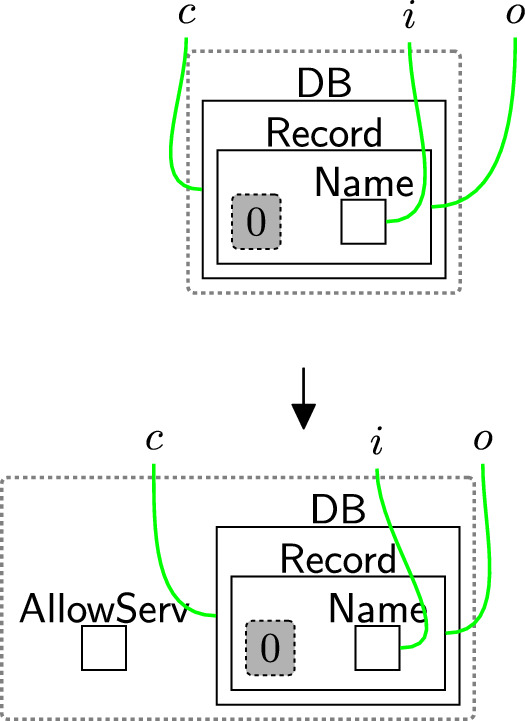
provideServices: once the user registers with the system (their name is stored), then they are able to start using the system’s services ($\textsf{AllowServ}$)

**Fig. 48:**
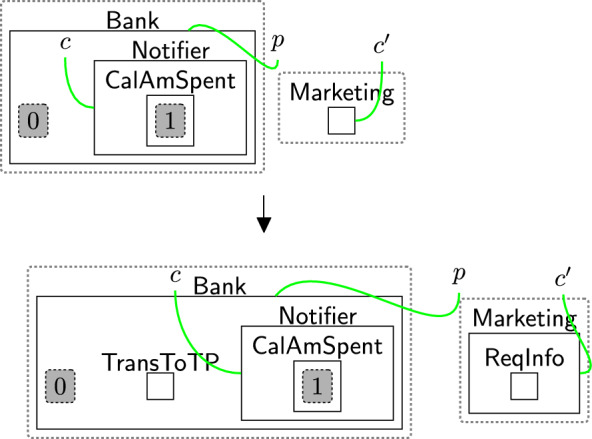
branchReqInfo: $\textsf{Marketing}$ requests the user’s transaction details through $\textsf{ReqInfo}$. The entity $\textsf{TransToTP}$ indicates that the system will transfer this data to $\textsf{Marketing}$

**Fig. 49:**
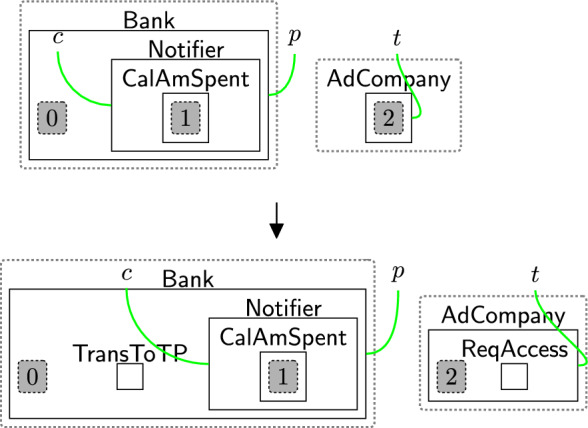
adCompReqInfo: $\textsf{AdCompany}$ requests the user’s transaction details through $\textsf{ReqAccess}$. The entity $\textsf{TransToTP}$ indicates that the system will transfer this data to $\textsf{AdCompany}$

**Fig. 53:**
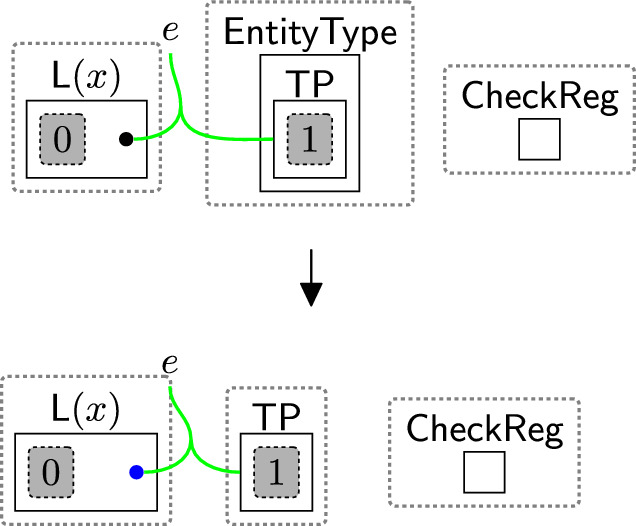
$\texttt {checkTPReg}(x)$: checking the region of the third party, whether it is a sender or receiver, by tagging its pointer, where *x* represents the name of a country or jurisdiction

**Fig. 54:**
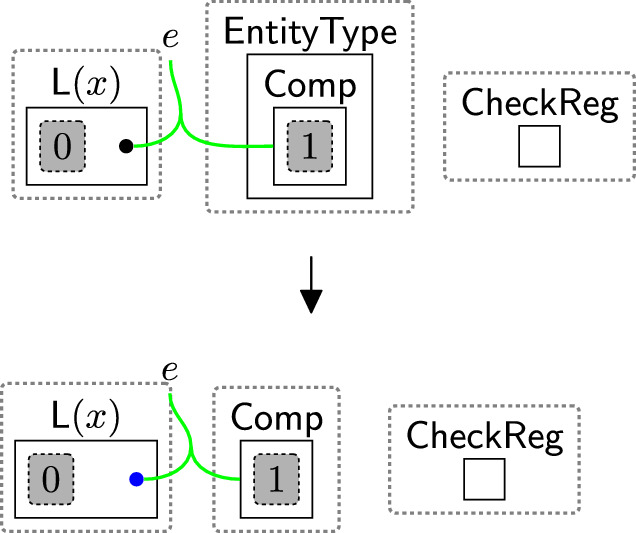
$\texttt {checkCompReg}(x)$: checking the region of components, whether they are senders or receivers, by tagging their pointers, where *x* represents the name of a country or jurisdiction

End-users could define their system-specific rules incorrectly, potentially leading to privacy violations. For example, Fig. 42a shows a system rule that transfers data from the database to the statistics department at the $\textsf{NRA}$. This is only valid if the user has accepted optional policies, but the rule has not checked consent before transferring the data. This means we might end up in the (partial) state shown in Fig. 42b where data has moved without a user’s consent.

This is detected by property (4) as the property is not fulfilled in this case. The end-users need to fix that by matching over the privacy perspectives, specifically, matching over $\mathsf {Comp\_F}$ to prevent sharing and $\textsf{Comp}$ to share the information as shown in Figs. 38 and 39. For more complex examples, a model checker will give a full trace that can aid experts in determining a suitable fix (and the model checker can then prove the fix was correct).

Although our framework primarily focuses on modelling the privacy notions we consider in this paper rather than directly addressing adversary models, the scenario in Fig. 42 illustrates how adversarial behaviour could be represented. While this scenario is examined through the lens of regulatory compliance, it highlights a situation in which bypassing consent checks could be considered adversarial. This violation can also be detected using property (4). Through the model checker, developers can adjust the system’s behaviour to prevent such actions, e.g. by triggering alerts if any attempt to bypass the consent check is detected and subsequently blocking $\textsf{StatDep}$.

### Static analysis

As we generalise the framework, we apply inductive reasoning to prove the correctness of the reaction rules^10^, thereby helping us ensure that the desired properties are preserved, even in the presence of state space explosion. Inductive reasoning proves that if a property holds for one state, it will continue to hold for all subsequent states, ensuring the property remains true throughout the system’s execution. Meanwhile, invariant reasoning identifies properties that remain unchanged throughout the system’s execution.

The correct application of the rules is tightly coupled to the priority classes that enforce the proper ordering of the rules. This means the analysis needs repeated for different regulations as these might change the rule priorities (Sect. 7). We use GDPR as an illustrative example.

The following analyses are proof sketches; a full formal proof would require more detailed examination, including all possible interleavings and conflict analysis.

**Property 1:** $\texttt{rejAll}$ does not lead to $\texttt{confirmation}$:

Rule rejectAll (Fig. 10) does not lead to the confirmation of the consent. When rule rejectAll (Fig. 10) is applied, it generates the entity $\textsf{Rejected}$. Rule confirm (Fig. 11) requires the entity $\textsf{Accepted}$ to be present on its left-hand side to be applicable. Since rule rejectAll (Fig. 10) produces the $\textsf{Rejected}$ entity instead of the $\textsf{Accepted}$ entity, the left-hand side of rule confirm (Fig. 11) cannot be matched. As a result, the system state that results from applying rule rejectAll (Fig. 10) cannot transition to a state where consent is confirmed. This ensures that the rejection of the consent does not eventually lead to its confirmation.

**Property 2:** No $\texttt{shareInfo}$ if $\texttt{rejectOpt}$:

This can be verified by visually examining the reaction rules. If $\mathsf {Comp\_F}$ appears on the left side of a rule, the user’s data must not be nested within the agent linked to $\mathsf {Comp\_F}$ on the right side of the rule. We can also use predicates to automatically verify this by searching for $\mathsf {Comp\_F}$ and checking if the agent linked to it contains the user’s data.

**Property 3:** $\texttt{invalidSCCs}$ does not lead to $\texttt{shareInfo}$:

By inspecting the initial state in Fig. 3, we can deduce that the $\textsf{SCCs}$ is invalid because it has a closed link. This implies that data should not be shared with the agent linked to a pointer nested within $(\texttt{ China})$, i.e. $\textsf{Marketing}$. We can verify this by examining the right-hand side of the rules to ensure that $\textsf{Marketing}$ does not access the user’s data, e.g. preventMDSCCs (Fig. 34).

**Property 4:** No $$\texttt{unauthAccess}$$:

We can also detect unauthorised access by inspecting the initial state and the right-hand side of the rules. By checking the initial state (Fig 3), we confirm that $\textsf{Notifier}$ is an unauthorised agent, as the $\textsf{Comp}$ linked to it does not linked to $\textsf{AuthAgent}$. Similarly, $\textsf{Name}$ is identified as personal information. We can prove that there is no unauthorised access because $\textsf{Name}$ is not nested within $\textsf{Notifier}$ or $\textsf{Marketing}$ on the right-hand side of the rules.

**Property 5:** $\texttt{withdAll}$ leads to $\texttt{deletingInfo}$:

$\textsf{DeleInfo}$ is generated by rule processWithdrawal (Fig. 19). This means whenever $\textsf{DeleInfo}$ appears on the left-hand side of a rule, it indicates that consent has been withdrawn. Consequently, the user’s data should not appear on the right-hand side of the rule. The right-hand side should not also contain any sites that might hold the user’s data, ensuring complete removal of the data post-withdrawal.

## Discussion

Collaboration between developers (who specialise in bigraphs) and privacy experts is essential for fully benefiting from the framework. While developers are responsible for applying bigraphs to ensure privacy compliance, privacy experts and policymakers do not directly engage with the technical model. Instead, they review the formal analysis and provide legal and regulatory guidance.

In certain instances, privacy experts or policymakers must comprehend specific technical dimensions of systems. However, the inherent complexity of these systems can impede their complete understanding and limit their capacity to offer informed feedback on privacy concerns [11]. To address this challenge, developers can utilise diagrammatic representations to depict the system’s structure and compliance with privacy regulations. This visual approach enables experts and policymakers to grasp the system’s behaviour without necessitating extensive immersion in technical details.

Regarding reusability, the framework includes 19 predefined privacy reaction rules. In the banking system example, all 19 rules are used, while the healthcare system example requires only 13 of these rules. This demonstrates the framework’s ability to adapt to different domains by reusing predefined rules. While additional case studies are needed, these examples show that the framework can be repurposed with minimal modification, highlighting its reusability.

Our framework abstracts system complexity, e.g. modelling a single user instead of multiple users, and excludes system-specific details like data storage or operations (e.g. calculating spending in the banking system example). As a result, system-specific processes and interactions must be considered when translating the model into the actual implementation, ensuring correct application of privacy regulations.

Priority classes categorise actions by importance, ensuring that high-priority privacy operations, such as consent withdrawal, are handled immediately, while lower-priority tasks continue seamlessly. This hierarchical management enables privacy checks to run concurrently with other activities, preventing system blocking and maintaining compliance without rendering the system unusable.

While this paper demonstrates the applicability of the proposed framework through two case studies, we recognise that scalability remains a significant challenge when applying the framework to real-world systems. Like many model-checking approaches, the state space can grow considerably when applied to large systems [46]. However, this is not an issue in our work, as we abstract away system-specific aspects and focus on modelling privacy regulations.

To provide an initial scalability assessment, we measured PRISM’s time and memory usage for the two examples. The bank model requires a Resident Set Size (RSS) of 131.2 MB, a Virtual Memory Size (VSZ) of 394.7 MB, and an execution time of 0.009 seconds. The second example requires an RSS of 129.9 MB, a VSZ of 404.8 MB, and an execution time of 0.002 seconds. These results indicate that PRISM’s time and space requirements are manageable for the models considered. Future work will extend this evaluation to larger systems to further assess scalability.

Informed consent has known limitations [47], e.g. users often fail to read privacy notices or fully understand data collection policies. Our approach can addresses this by presenting verification results, e.g. formal certificates or regulatory approvals [48], to assure users that the system operates in accordance with regulations.

The framework promotes transparency by visually representing data flows and system behaviour, enabling users and designers to understand how data is used and shared. This can foster trust in privacy practices by reassuring data owners that their data is handled according to regulations, even beyond their immediate control. However, complete adherence to all privacy principles requires modelling and rigorous examination of each principle. The framework only guarantees compliance with the privacy aspects addressed in this paper.

The proposed framework generates a model that captures the privacy concepts we consider in this paper. However, the interaction between our privacy model and the system model defined by the developers could generate bugs affecting privacy, such as those produced by conflicts between the privacy and system rules. Conflicts may arise due to improper handling of priority classes. For instance, if we assign a lower priority to rule closeLinks than to rules confirm and registerRequest, this could result in rule registerRequest being executed after confirm, causing closeLinks to be skipped and not executed.

**Table 4:** Comparison of formal methods for modelling privacy regulations

Approach	Regulation	Automated verification	Verification type	Spatial properties	Cross-border transfer	Diagrams
PrivacyAPIs [49]	HIPAA	$\checkmark$	D	$\times$	$\times$	$\times$
MBIPV [50]	GDPR	$\checkmark$	D	$\checkmark$	$\times$	$\checkmark$
Isabelle/HOL [51]	GDPR	PA	ThP	$\times$	$\times$	$\times$
Hublet [52] et al.	GDPR	$\checkmark$	D	$\times$	$\times$	$\times$
CI [53]	HIPAA, COPPA, GLBA	(-)	(-)	$\times$	$\times$	$\times$
Model-driven [54]	GDPR	(-)	(-)	$\times$	$\times$	$\times$
PrivacyLFP [55]	HIPAA, GLBA	(-)	(-)	$\times$	$\times$	$\times$
DPL [56]	GDPR	PA	D/ThP	$\times$	$\times$	$\times$
P-AOL [57]	GDPR	$\checkmark$	D	$\times$	$\times$	$\times$
Privacy calculus [14]	GDPR	(-)	S	$\times$	$\times$	$\times$
DiálogoP [15]	GDPR	(-)	(-)	$\times$	$\times$	$\checkmark$
Bigraphical Framework	GDPR, CCPA	$\checkmark$	D/S	$\checkmark$	$\checkmark$	$\checkmark$

*PA* partially automated, *D* dynamic, *S* static, (−) not mentioned, *ThP* theorem proving, *HIPAA* the Health Insurance Portability and Accountability Act, *COPPA* the Children’s Online Privacy Protection Act and *GLBA* the Gramm–Leach–Bliley Act

Linking system entities to permissions for defining initial states is challenging, as it may result in mislinked permissions. For example, if we incorrectly link $\textsf{Ad}$ and $\textsf{OptIn}$ in Fig. 3 with a $\textsf{Comp}$ other than the one linked to $\textsf{Marketing}$, closeLinks will close the link of the wrong $\textsf{Comp}$. This leads to sharing data with $\textsf{Marketing}$, even if the user has rejected sharing their data as the $\textsf{Comp}$ linked to $\textsf{Marketing}$ is not changed to $\mathsf {Comp\_F}$.

The developers need to use PRISM to *automatically* detect these bugs, which can then be fixed accordingly. To proactively address these potential issues, we have pre-defined a set of privacy properties for end-users to use directly, eliminating the need for them to define these properties themselves. This not only simplifies the process but also significantly reduces the risk of errors by the developers when defining privacy properties, ensuring the accuracy of the model.

## Related work

Model checking is widely used to verify privacy specifications. Behaviour-aware privacy [58], PILOT [59], and PrivacyAPIs [49] employ SPIN and LTL, focusing on restricted access, user-defined policies, and HIPAA compliance, respectively. Joshaghani and Mehrpouyan [60] propose a model-checking framework for user-defined policies, while Ye et al. [50] leverage NuSMV [61] and CTL for GDPR compliance.

Other researchers adopt theorem proving. Kammueller [51] and TTC [62] rely on Isabelle/HOL to prove GDPR compliance in IoT healthcare and verify privacy by design, while S4P [63] and SIMPL [64] employ trace semantics for policy enforcement. Hublet et al. [52] use metric first-Order temporal logic, and frameworks like OSL [65], Rei [66], CI [53], PrivacyLFP [55], and model-driven privacy [54] apply first-order or temporal logics for compliance.

P-AOL [57] extends an active object language with privacy constructs, with a Maude-based prototype [67] that validates GDPR consent. DPL [56] also targets GDPR via multiset rewriting in Maude, though partial manual proofs are required. QPDL [68] augments LTL with spatial concepts but lacks support for dynamic changes. Jeeves [69] uses the $\lambda$-calculus [70] to enforce developer-specified privacy rules.

Since data mobility is crucial, many works adopt the $\pi$-calculus [71]: Mancini [72] extends ProVerif [73] to handle unlinkability, while Kouzapas and Philippou [74] propose a type-checking privacy calculus later enhanced by Vanezi et al. [14] for GDPR and extended into Di’alogoP [15] with multiparty session types [75].

Formal methods often offer limited support for cross-border data transfers, though some *informal* approaches exist. For example, Guamán et al. [76, 77] and Hunter [78] provide informal or machine learning-based analyses for international transfers. Unlike formal methods, these approaches provide a less rigorous analysis of the GDPR requirements for cross-border data transfers.

Table 4 shows that many approaches rely on dynamic verification, which handles real-time updates, e.g. cross-border data transfers or consent modifications. By contrast, static verification keeps the system configuration fixed throughout analysis. The table also highlights that few methods offer diagrammatic support and capture spatial properties. The presented approaches are also not developed to address cross-border transfers. Methods not shown in the table are not explicitly tailored to privacy regulations, lacking constructs for real-time modifications and international transfers [79].

Our bigraphical framework fills these gaps by: (1) supporting both static and dynamic verification, (2) performing automated CTL-based checks in PRISM, (3) providing diagrammatic representations, and (4) offering explicit spatial modelling to handle cross-border data transfer requirements.

## Conclusion

Ensuring privacy is a major concern for business and is complicated by the range of use cases and textual, non-formalised, regulations, e.g. GPDR and CCPA.

We have shown how to formally model privacy policies in a diagrammatic notation, based on bigraphs, that makes them amenable to automated verification through model checking. We believe the visual nature of the approach makes it suitable for a wide audience such as privacy experts who may not have the background knowledge to use complex mathematical modelling tools, e.g. those based on $\pi$-calculus or theorem proving. The approach is *reusable* and can capture multiple case studies, e.g. banking and healthcare examples, and *extensible* with the user being able to add/amend reaction rules to respond to changes in the underlying system or the regulatory environment.

In future, we will extend the model to support multiple users, allowing us to model violations where data is, for example, sent to the wrong person. The framework needs to be applied to complex case studies and investigate how the approach scales to these systems. We will also conduct user studies to evaluate the usability of the framework for system designers and privacy experts.

As bigraphs support using probabilities [80], the model can be extended to capture concepts such as *differential privacy* [81] by computing the probability of private information leakage when aggregate data about a group of users is shared with third parties.

We also plan to reuse the framework to model other regulations, such as the PDPL, GCDPA, APPs, and ADPPA, and extend it to capture common privacy principles across them, such as storage limitation (i.e. ensuring data is not retained once its processing purpose is fulfilled and, if no longer needed, should be deleted or anonymised).

Finally, the tooling will be improved to automatically generate valid initial states to reduce the risks of incorrectly linking permissions. This might be based on a sorting discipline (like a type system) that checks the correctness of the bigraphs [82, 83], or by developing new domain-specific languages which target bigraphs for further analysis.

## Figures and Tables

**Fig. 43:**
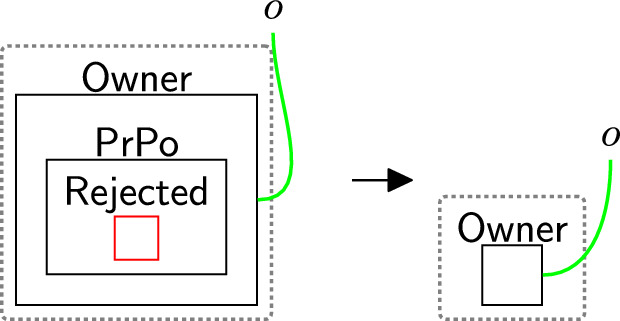
changeDecisions: as the data owner could change their mind on policies in the future, this rule is used to reset the bigraph to a state where rule sendPolicy (Fig. 4) can be applied again

**Fig. 45:**
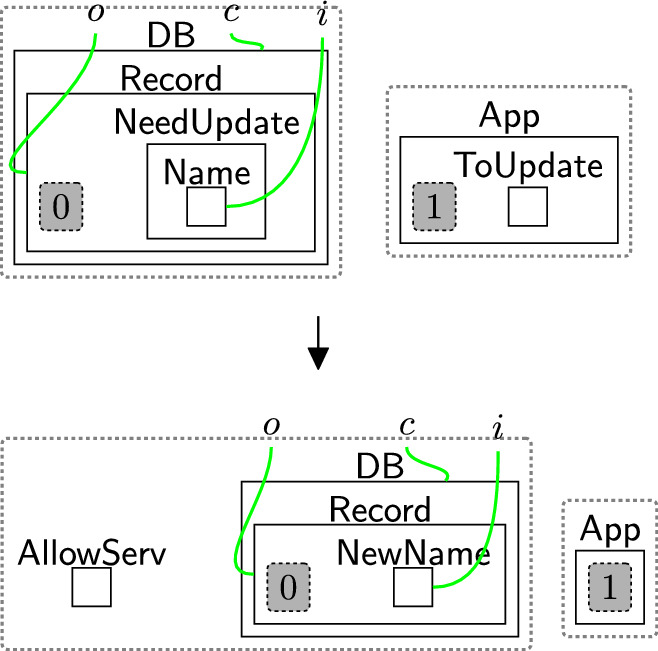
updatingName: the system updates the user’s name by replacing $\textsf{Name}$ with $\textsf{NewName}$. Note that the new name should have the same permissions of the previous name as it is derived information. The entity $\textsf{AllowServ}$ is generated again to allow the user to ask for a new service

**Fig. 46:**
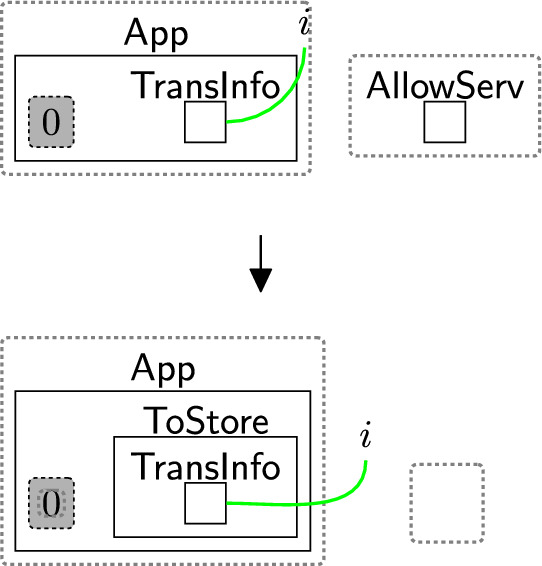
generateTrans: when the transaction is generated, it should be stored in the $\textsf{DB}$. The entity $\textsf{ToStore}$ is created to initialise the storing process in the $\textsf{DB}$

**Fig. 47:**
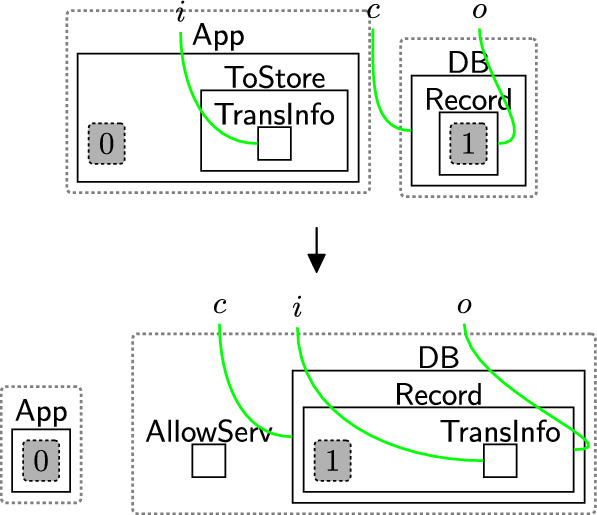
storeTransInfo: the system stores the generated $\textsf{TransInfo}$ in the user’s $\textsf{Record}$. To allow the user to ask for a new service, $\textsf{AllowServ}$ is created

**Fig. 50:**
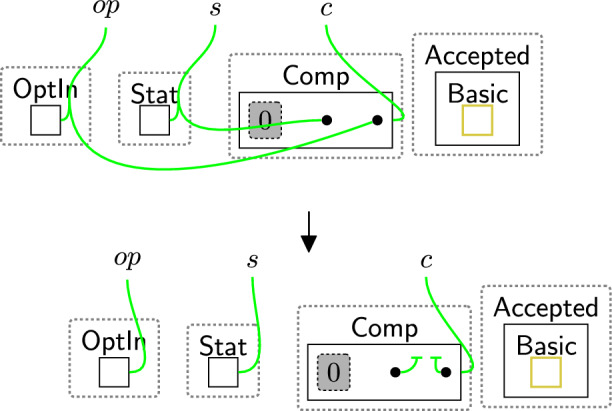
closeLinks: this rule is applied when the user requests to opt out (stop sharing their data with the statistical department at the NRA). It closes the links to the optional permissions, indicating that the user withdraws them

**Fig. 51:**
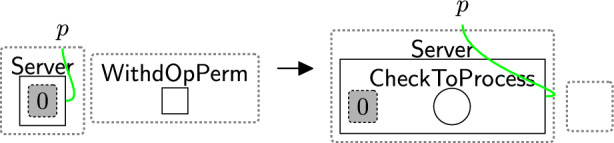
checkConOptOut: after withdrawing the optional permissions, this rule generates the entity $\textsf{CheckToProcess}$ to initiate the checking process of the permissions, allowing rule startCheck (Fig. 12) to be applied. This, in turn, changes the type of the third-party components to $\mathsf {Comp\_F}$ (changeTypeComp, Fig. 13). By doing so, any rule that shares data must match over $\mathsf {Comp\_F}$ to ensure that the data is no longer shared following the user’s request to stop sharing their data as shown in Fig. 39

**Fig. 52:**
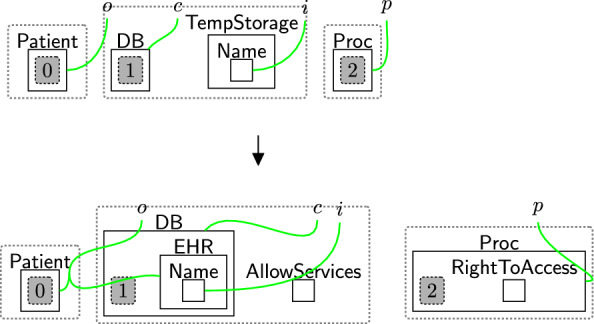
storingName: as the CCPA considers the patient to have consented to share their data, the patient’s name is stored directly once the user registers with the system. Unlike rule storingName Fig. 24, where the entity $\textsf{CheckPolicy}$ is replaced with $\textsf{RightToAccess}$, this rule does not include $\textsf{CheckPolicy}$ since no consent is required to check. Therefore, it generates the entity $\textsf{RightToAccess}$ to allow rule rightToAccess (Fig. 14) to be applied

## Appendix A: Additional rules for the banking example

We present additional reaction rules for the banking example. These rules outline the main system-specific processes, e.g. updating the user’s name, generating, and storing transactions etc(Figs. 43, 44, 45, 46, 47, 47, 48, 49).

## Appendix B: Additional system rules for the healthcare example

The following illustrates the diagrammatic notations of some system-specific rules used to model the healthcare example based on the California Consumer Privacy Act (CCPA) (Figs. 50, 51, 52):

## Appendix C: Checking regions

The following rules are used to check the region of third parties or components within the system (Figs. 53, 54):

## Appendix D: Algebraic definition of the privacy rules

This appendix presents the algebraic definitions (equivalent to the diagrammatic notations) for some of the privacy rules discussed in this paper. The definitions for the remaining rules can be found in [30].

In the following definitions, we use $\eta$ to denote instantiation maps.

## Footnotes

1 In general this can be any bigraph, but is often a single entity.

2 Since Monzo policies intentionally lack technical details to make them more accessible to non-technical users, we inspire these system-specific entities from [14].

3 We use the term *owner* to generalise across different privacy frameworks, such as *data subjects* in the GDPR and *consumers* under the CCPA.

4 We do not need to model the content of the SCCs, as they are standardised contractual clauses provided by the European Commission for data transfers to non-adequate countries.

5 To enable users to revise their decisions after the rejection, we can define a rule that resets the bigraph to a state where rule sendPolicy can be reapplied (see Fig. 43 in Appendix A).

6 The system needs rules to handle optional permissions etc. These rules are presented in “Appendix A”.

7 Similar to Monzo’s privacy policies, Fitbit’s privacy policies do not include technical details. As such, we adapt the system’s aspects from [44].

8 Since Fitbit does not explicitly state whether they employ notice at collection or obtain consent, we assume they adhere to the notice at collection practice as outlined in the CCPA.

9 Although we capture key features of privacy regulations, we cannot claim full adherence unless *every* aspect, e.g. retention time and data encryption, was fully modelled and checked, but we can operate with increased confidence.

10 Currently there is no tool support for this and it must be done by hand.
